# Implementation and experimental evaluation of mint for workload-aware approximate nearest neighbor index tuning in multi-vector databases

**DOI:** 10.1016/j.mex.2026.104151

**Published:** 2026-09-06

**Authors:** Sharda Yashwant Salunkhe, Kuldeep Vayadande, Hridaynath Pandurang Khandagale, Pavitha Nooji, Mangesh Hajare, Krishna Prakasha, Rakhi Bhardwaj, Manasi Phand, Anish Katariya, Ninad Kale, Amey Kharade

**Affiliations:** a Sharad Institute of Technology College of Engineering, Yadrav, India; b Vishwakarma Institute of Technology, Pune, India; c Department of Technology, Shivaji University, Kolhapur, India; d Vishwakarma University, Pune, Maharashtra, India; e Army Institute of Technology, Pune, India; f Manipal Institute of Technology, Manipal Academy of Higher Education, Manipal, Karnataka, 576104, India

**Keywords:** Vector databases, Approximate nearest neighbor search, Multi-vector search, HNSW, Index optimization, Workload-aware indexing, Query planning

## Abstract

Approximate nearest neighbor (ANN) search is an integral part of contemporary vector databases; however, the performance of this algorithm highly depends on the specifics of data, recall requirements, storage capabilities, and query workload. All these factors complicate ANN even further in multi-vector databases, where every record has several vectors and queries may utilize arbitrary combinations of vector fields. This paper provides an independent implementation and experimental analysis of MINT (Multi-Vector Search Index Tuning) framework, originally introduced in [9], for workload-aware ANN index tuning in multi-vector databases. The implementation follows the core workflow of MINT that includes workload-induced candidate index generation, cost and recall estimation of generated indexes through sampling, extended-k query planning, and configuration search under constraints of required recall and storage capabilities. The system was implemented in Python with usage of NumPy, hnswlib, scikit-learn, SciPy, and h5py, constructing HNSW indexes only after selecting a proper configuration. The experiments were conducted for a multi-vector database, composed of six columns based on GloVe, SIFT, Deep1M, and Music100 collections. The present study is focused on reproducibility at the implementation level, analysis of the tuning process and its assumptions, and comparison of workload aware configurations with baselines. The quantitative results obtained for the benchmark pipeline need to be considered separately from those of the optimization pipeline since the two execution pipelines have different experimental setups that include sampling fraction, recall threshold, top-k value, workload setup, and other restrictions; hence, benchmarking results cannot be viewed as the outputs of the optimization pipeline.

## Graphical abstract

**Figure:**
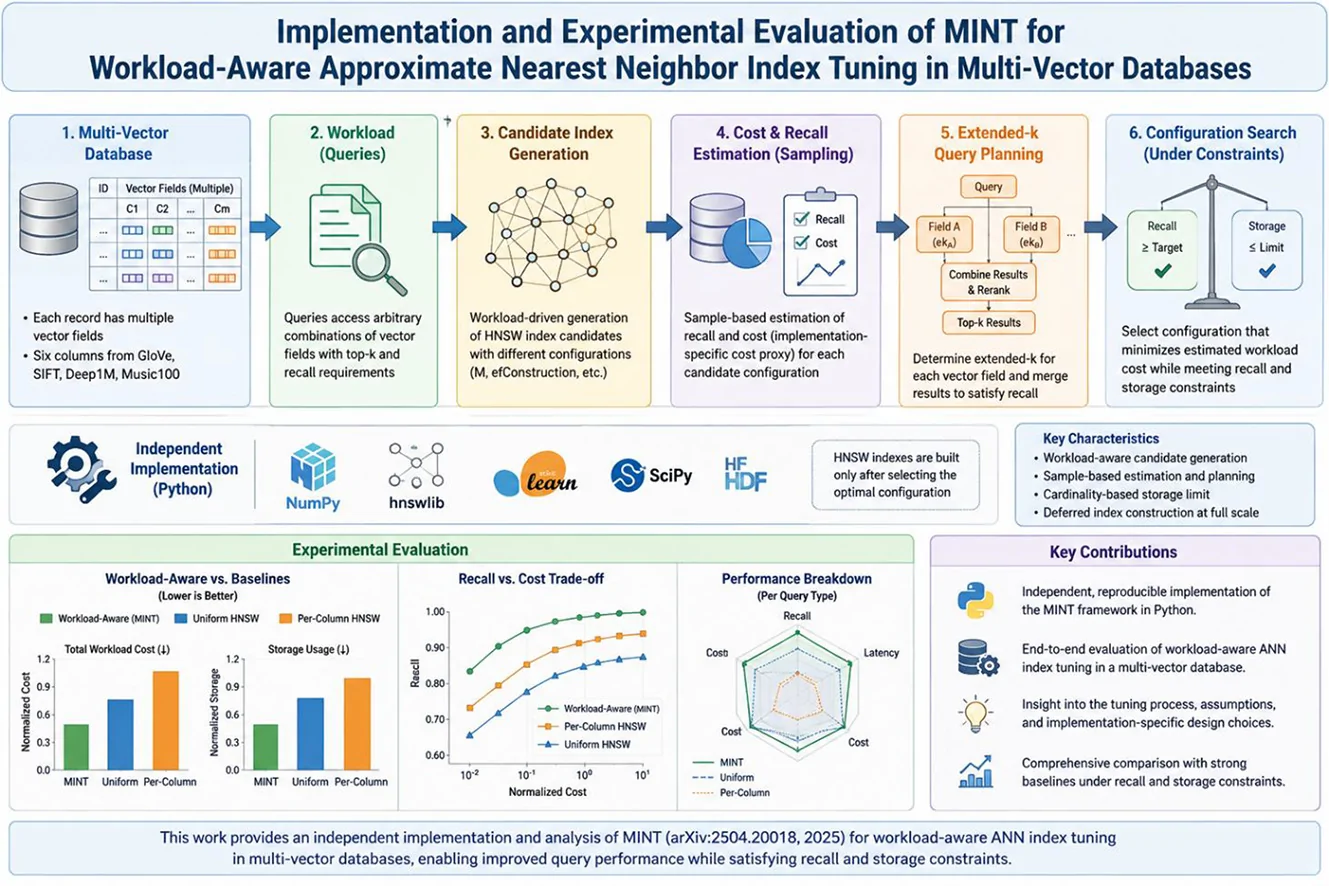

## Specifications table

**Table:** 

**Subject area**	Computer Science
**More specific subject area**	Vector Databases, Approximate Nearest Neighbor (ANN) Search, Multi-Vector Retrieval, Query Optimization, Machine Learning Systems
**Name of your method**	Independent implementation and evaluation of MINT (Multi-Vector Search Index Tuning), based on the MINT framework introduced in [1]
**Name and reference of original method**	**Original method:** MINT (Multi-Vector Search Index Tuning), introduced in [1]. Zhu, J., Wang, Y., Ding, B., Bernstein, P.A., Narasayya, V., Chaudhuri, S., “MINT: Multi-Vector Search Index Tuning,” arXiv:2504.20018, 2025. MINT framework defines multi-vector index tuning as the selection of ANN index configurations and queries that minimize workload cost under the constraint of recall and storage limits. Workload-driven generation of candidates, sample-based cost and recall estimates, extended-k query planning, and configuration search form the core of the MINT approach. This study does not pretend to introduce MINT as a new algorithm. Instead, it is a separate Python implementation and implementation-centric experimental assessment of the MINT methodology. Implementation utilizes HNSW via hnswlib as a physical representation of ANN indexes and makes several adjustments and adaptations specific to implementation, such as cardinality-based storage limit, planning behavior based on samples, implementation-specific cost proxy, and deferred construction of HNSW indexes at full scale. At the same time, the implementation relies on well-known ANN algorithms and techniques, such as HNSW [2], whereas IVF, PQ, and DiskANN are discussed as alternative ANN index families.
**Resource availability**	**Source code:** The implementation used in this study is available at “https://github.com/1Ninad/vector-index-optimizer”. The repository contains workload generation, estimator training, candidate generation, query planning, configuration search, HNSW construction, serving, benchmark utilities, and automated tests. **Programming Language:** Python 3.11+ **Libraries:** NumPy, hnswlib, Scikit-learn, SciPy, h5py **Datasets:** GloVe word embeddings (50d, 100d, 200d), SIFT image descriptors, Deep1M image features, Music100 audio embeddings **Hardware:** Standard CPU-based environment (hardware specifications not fixed in repository) **Software Requirements:** Python environment with required packages listed in requirements.txt

## Background

Embeddings in vector form support tasks like finding related content, suggesting items, searching across media types, or enhancing responses using retrieved data. These approaches turn items into points within a space that has many dimensions. Finding matches means quickly identifying nearby points in vast sets of such representations. Because pinpointing exact neighbors becomes too slow when dealing with massive volumes, approximated techniques step in - sacrificing minimal accuracy to achieve much faster performance [3,4].

Yet ANN effectiveness depends closely on how indexes are built, what settings they use, available memory, along with patterns of incoming queries. Instead of uniform structures, graph strategies like HNSW achieve high accuracy when data fits in main memory. In contrast, techniques splitting space or compressing vectors - IVF and PQ, for example - balance speed against resource demands more efficiently [2,5,6]. When moving storage out of RAM, approaches like DiskANN can be used to adapt nearest neighbor searches for data stored on disk [7]. Yet, there seems to be no universal way as behaviors change based on load conditions and constraints of the underlying system architecture [8,9].

In cases where several vector columns are related to each entry (due to different embedding or types of information), the task becomes very complicated for a multi-vector database scenario. A query may involve only one vector column or multiple ones at the same time, and each case requires a different approach to be taken into account when utilizing indexes. Selecting one type of indexing strategy is usually not an optimal choice in such situations, and the key challenge is to figure out which indexes to use, with what combination of vectors and query methods.

Modern vector database systems use different ANN indexing families, including graph-based, quantization-based, disk-based, and hybrid approaches. This diversity motivates the need for workload-aware index selection instead of relying on a single fixed indexing method.

This study implements the MINT (Multi-Vector Search Index Tuning) framework originally introduced in [1]. MINT addresses multi-vector index tuning as a workload-driven optimization task, where a certain index configuration and respective query plans are chosen under the constraints of recall and storage. MINT comprises workload-driven candidate generation, sampling-based estimation, extended-k query planning, and configuration search. The contribution of the present work is the independent Python implementation, reproducibility-oriented documentation, implementation-specific adaptations, and experimental examination of the MINT workflow introduced in [1]. The present implementation follows the principal MINT workflow and investigates its behavior in a repository-driven experimental setup based on a six-column vector database. The present implementation consists of modular Python components for workload generation, estimator training, candidate-index generation, query planning, beam-search configuration selection, HNSW index construction, and serving-time candidate reranking. A number of features of the implementation are different or simpler than those in the original formulation. For example, the physical ANN indexes are implemented using HNSW through the hnswlib library; the storage restriction is expressed by the number of indexes used as opposed to byte level storage measurement; the cost estimator in the implementation is based on an extended-k proxy, rather than instrumentation of the internal distance computation performed by HNSW; and plan recall is determined based on retrieval overlap on sampled data, rather than solely on the learned recall model.

The current literature has started looking at vector search optimization from a workload and system perspective as opposed to viewing ANN indexing as an algorithmic problem in isolation. The original MINT study [1] defined the problem of index tuning for multi-vector search workloads as the problem of choosing the indexes and query plans which would minimize the workload latency while meeting the requirements on recall and storage. The MINT framework in [1] included aspects like workload driven candidate generation, sampling-based cost and recall estimation, extended k-query planning, and configuration search for multi-vector database systems. Even the study on filtered vector search indicated that a collection of workload-specific indexes was able to perform better than having a single universal index, especially in cases where the predicate forms and selectivity’s were different. Unified execution over multiple vector indexes has also been explored in systems with multi-index retrievals.

Built around a real repository-driven workflow, the system starts by loading a six column vector store. Following that, it either pulls in existing workloads or creates artificial ones. From there, sampling the data feeds into training basic statistical models. Then come trial index structures along with initial setups generated as options.

**Fig. 1:**
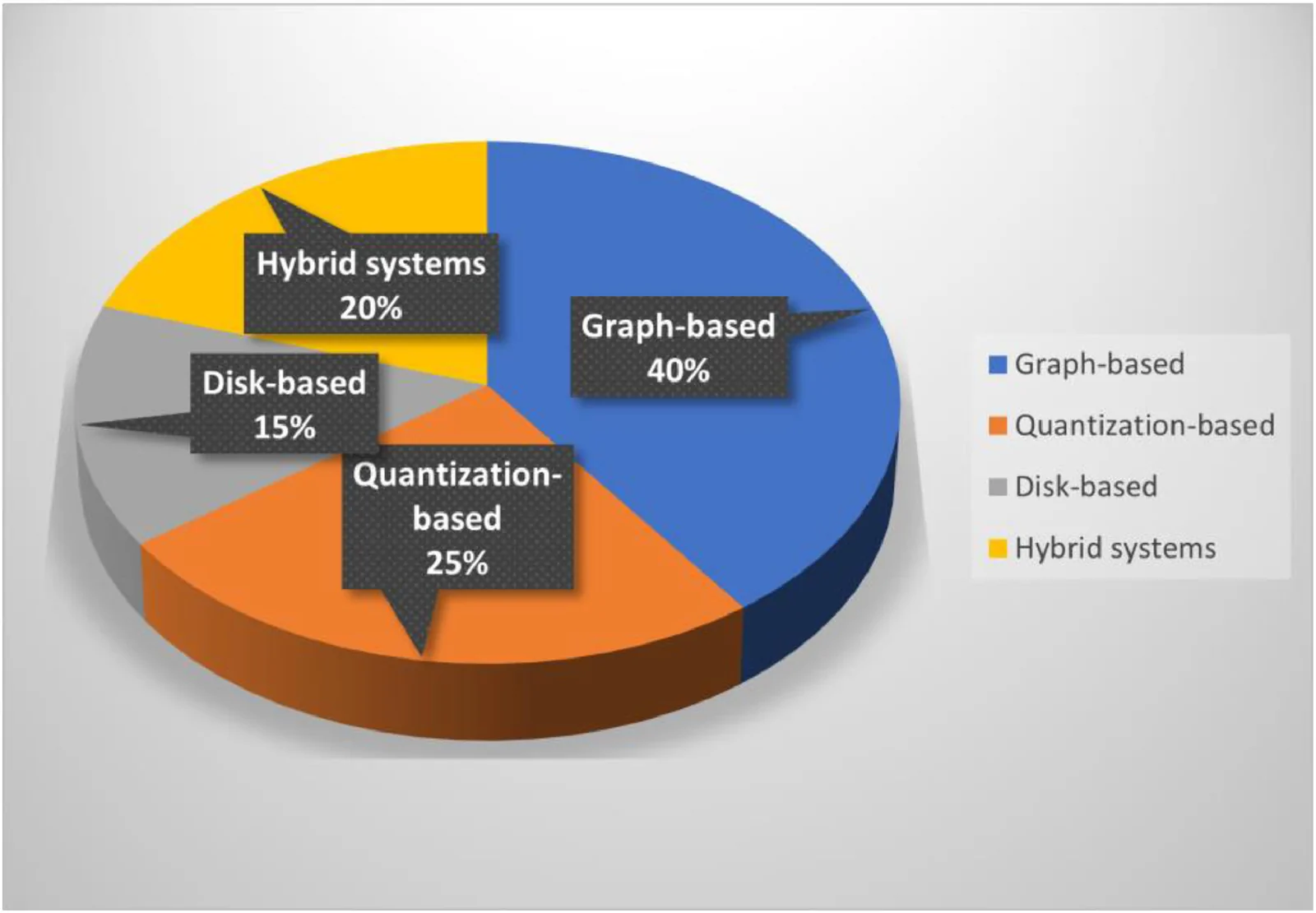
Distribution of major ANN indexing categories considered in modern vector database systems.

Fig. 1. summarizes the major ANN indexing categories considered in modern vector database systems.

A stepwise selection process, beam search picks one setup based on exploration depth. Only after choice does actual construction occur, limited to selected HNSW variants. Queries later execute through merged results refined in sequence. This hands-on structure matters not just as theory. One reason lies in tying every idea to working code components instead of abstract diagrams. Other stems from pushing a clean split: predictions made mid search stay separate from performance logs saved back into storage.

The main contributions of this work are as follows:

• An independent implementation of the principal MINT workflow for workload-aware index tuning in a multi-vector database setting.
• A modular Python implementation covering workload generation, candidate index construction, sample based estimator training, query planning, beam-search configuration optimization, HNSW index construction, and serving time reranking.
• An implementation level analysis that documents where the present system follows the original MINT methodology and where implementation specific simplifications or adaptations are introduced.
• An experimental evaluation of the implemented tuning pipeline using a six column database assembled from heterogeneous vector datasets.
• A reproducibility oriented description of parameter settings, estimator behavior, configuration search, selected index construction, and evaluation methodology.
• An explicit analysis of implementation limitations, including the cost proxy, cardinality based storage model, sample based recall evaluation, and differences between optimization time estimates and measured serving behavior.

## Relation to the original MINT framework

This study is therefore presented as an independent implementation and evaluation of the MINT framework introduced in [1], rather than as the introduction of a new index tuning methodology. The present implementation preserves the principal components of MINT, including workload driven candidate-index generation, extended-k query planning, sample-based characterization of retrieval behavior, configuration optimization under recall and storage constraints, and delayed construction of selected ANN indexes.

The present implementation is not an exact reproduction of the original system. It was developed independently in Python and specializes physical ANN indexing to HNSW through the hnswlib library. In addition, storage is represented by the number of selected indexes rather than byte-level storage consumption, the implemented cost model uses extended-k as a proxy for distance computation effort, and query plan recall is evaluated against ground truth on sampled data. These differences are treated as implementation specific adaptations and limitations.

## Method details

Following [1], this section describes how the multi-vector index-tuning problem is realized in the present implementation. The mathematical formulation and core optimization concepts are adopted from the MINT framework, while implementation specific behavior and adaptations are identified separately. This section describes the multi vector index tuning problem implemented in the present system and aligns the mathematical notation with the corresponding implementation components.

### Multi-vector database model

Each item in the database holds several vector columns. Every such column ties to a specific kind of embedding, marked through an integer identifier. When referring to selected identifiers, the symbol vid stands for those particular columns involved in either search or indexing. Inside the system, an Index structure keeps track of that fixed group along with the full size obtained by joining their vector data arranged sequentially. The mathematical characteristics, strengths, and limitations of the principal ANN indexing methods considered in this study are summarized in Table 1.

**Table 1:** Mathematical foundations of ANN indexing methods.

Method	Core Equation	Complexity	Strength	Limitation
HNSW (Graph-based)	$d\left(q,x\right)=∥q-x{∥}_{2}$ with greedy graph traversal	*O* (log *N)* average	Strong recall-quality trade-off	Memory-sensitive tuning
IVF (Partition-based)	*x* → *Ci, d*(*q, x*) ≈ *d*(*q, Ci*)	Probe - dependent	Search - space pruning	Partition miss risk
Product Quantization (PQ)	*x* ≈ [*q*1(*x*)*, q*2(*x*)*, ..., qm*(*x*)]	Codebook - dependent	Compact Vector Representation	Approximation error
DiskANN	*d*(*q, x*) with disk-aware graph traversal	Search + I/O cost	Extends ANN to larger storage tiers	I/O sensitivity
Learned Index	*f* (*q*) → candidate set	Model - dependent	Predictive adaptation	Training and maintenance overhead
Hybrid Systems	*Score* = *α*· *ANN* +*β* · *Filter*	Query - dependent	End-to-End query integration	Optimization complexity

The notation used throughout the optimization formulation is defined in Table 2.

**Table 2:** Notation used in the optimization problem.

Symbol	Meaning
(c)	Integer identifier of one vector column in the database
(vid)	Set of vector-column identifiers referenced by an index or query
(x_i_)	One candidate ANN index
(X)	Index configuration, i.e., a set of selected indexes
dim(x_i_)	Total vector dimension of index (x_i_)
(q)	One query
vid(q)	Set of vector columns referenced by query (q)
dim(q)	Sum of the vector dimensions referenced by query (q)
(k)	Number of final nearest-neighbour results returned for a query
(ek_i_)	Extended-(k) value assigned to index (x_i_); (ek_i_=0) means that the index is skipped
(EK_q_)	Set of extended-(k) assignments selected for query (q) under configuration (X)
gt(q)	Exact brute-force top-(k) ground-truth result set for query (q)
(\theta_{\mathrm{recall}})	Minimum recall threshold required for a feasible query plan
(\theta_{\mathrm{storage}})	Maximum number of indexes allowed in a configuration
P(q)	Probability weight of query (q) in workload (W)

The representative recall latency and memory recall trade-offs across ANN indexing techniques are illustrated in Fig. 3, Fig. 4, respectively.

### Query and workload model

Following [1], the present implementation represents the workload as a probability weighted collection of multi-vector queries and uses the same workload/configuration concepts as the original MINT formulation. A single query *q* points to at least one target column, tied to a vid, includes a vector per chosen column, specifies a top-*k* number, along with an overall query dimension. The broader classification of graph based, quantization-based, and disk-oriented ANN indexing families is illustrated in Fig. 5.

Found within groups of queries paired with likelihoods forming a workload here, every probability adjusts to scale, together reaching exactly 1:

$$\sum_{q\in W}P\left(q\right)=1$$

The implementation stores the workload as a list of WorkloadEntry objects.

### Configuration model

A setup *X* contains possible index positions. Given any search *q* along with setup *X*, the method picks a strategy showing which indices within *X* to examine alongside an adjusted-*k* figure per chosen index. Only when matching conditions are met can an index support a query

vid(x)⊆vid(q)

When the number of columns falls short, exclusion happens - driven by *DI*, shaping what fits through the size-gap filter. The system moves on, leaving behind those that do not meet the threshold defined by this measure.

### Optimization objective

Following [1], the optimization objective implemented in this work is to minimize probability weighted average workload cost while satisfying the per query recall requirement and the configuration storage constraint. Given a workload W, a recall threshold θ_recall_, and a storage budget θ_storage_, the objective is

$$\setminus min_{X}\sum_{q\in W}P\left(q\right)\cdot cost_{\left(plan\right)\left(q,X,EK_{q}\right)}$$

subject to

$$recall\left(q,X,EK_{q}\right)\geq \theta_{recall}\setminus quad\forall q\setminus in\;W$$

and

$$\left|X\right|\leq \theta_{storage}$$

### Cost model

Following the MINT cost-model structure in [1], the present implementation uses estimated cost as an optimization proxy rather than as a direct predictor of wall clock query execution cost. For an index *x_i_* and extended-*k* value *ek_i_*, the index scan cost is

$$cost_{ijx}\left(q,x_{i},eki\right)=x_{i}\cdot \dim\cdot numDist\left(q,x_{i},eki\right),$$

where *x_i_.*dim is the total dimension of the index and numDist is the estimated number of distance computations.

Upon obtaining candidates from the chosen indices, the obtained candidates are merged and re-ranked based on the entire query vector. The cost of reranking is

cost_rerank(q,EK)=q.dim×Σ_iek_i

The total plan cost is therefore

cost_plan(q,X,EK)=Σ_icost_idx(q,x_i,ek_i)+cost_rerank(q,EK)

The above costing model is consistent with the process in which the planner works: it uses index size to compute the cost of scanning and adds the extra element for ordering based on query and candidate counts.

### Recall model

Following the recall definition in [1], for a query *q*, recall is the ratio of the ground-truth top-*k* items that are found by combining the results from the selected indexes:

recall(q,X,EK)=|gt(q)∩⋃_ires(q,x_i,ek_i)|/|gt(q)|

where *gt*(*q*) denotes the ground-truth top-k result set obtained using brute-force search, and

*res*(*q, x_i_, ek_i_*) denotes the results set obtained using index *x_i_* with extended-*k* value *ek_i_*.

### Storage model

Following the storage-constraint formulation in [1], the present implementation uses a cardinality-based storage model:

storage(X)=|X|

For this reason, limits apply to chosen indexes by count, not memory footprint, data links, or byte volume. Intentionally kept simple, it mirrors how the system calculates storage needs.

### Optimization scope

This implementation solves a static problem of index configuration. Issues related to online tuning with respect to changing workload, attribute based filtering, distribution of execution, and vector relational hybrid querying are beyond the scope of this research. They will be elaborated upon in the limitations and future research sections.

**Fig. 2:**
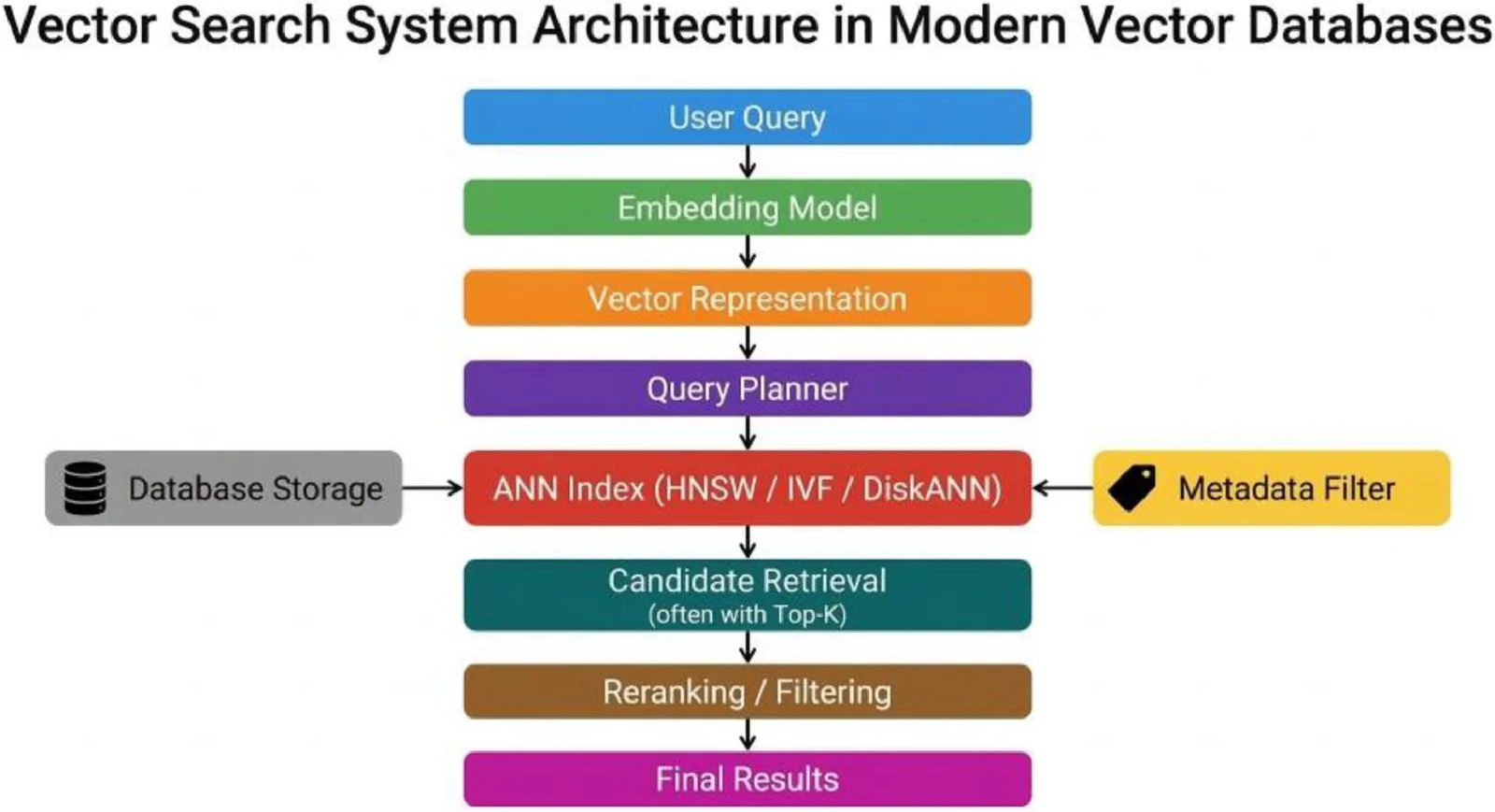
System level workflow of the present MINT implementation, including workload analysis, estimator training, configuration search, and final HNSW index construction.

Starting with how workloads are represented, the method moves step by step toward building the final index, guided by the optimizer pipeline shown in Fig. 2. Instead of remaining abstract, the approach is backed by a repository that enables both high-level explanation and precise alignment with implemented algorithms.

**Fig. 3:**
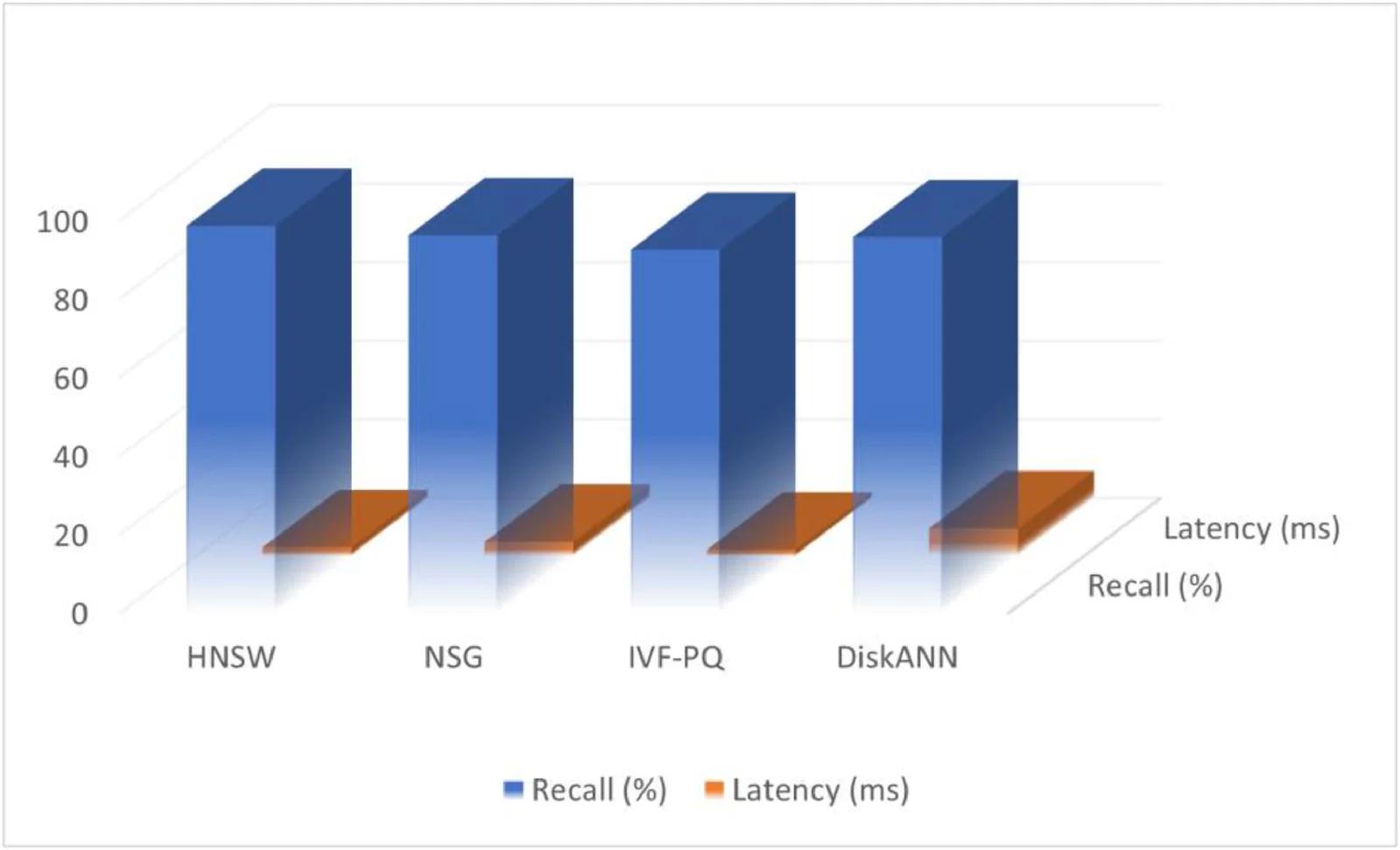
Comparison of recall and latency across representative ANN indexing techniques.

**Fig. 4:**
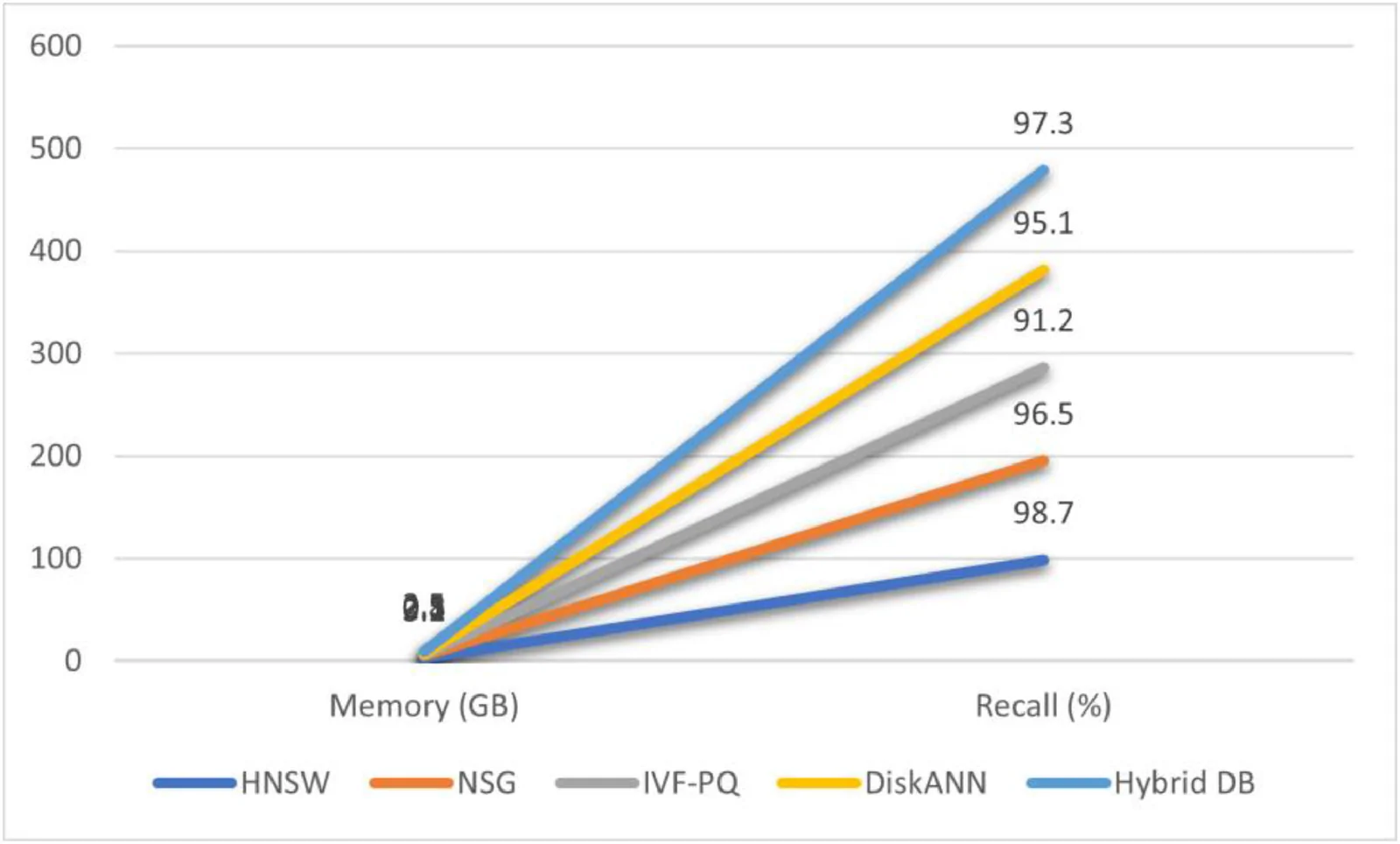
Memory usage versus recall across representative ANN indexing methods.

**Fig. 5:**
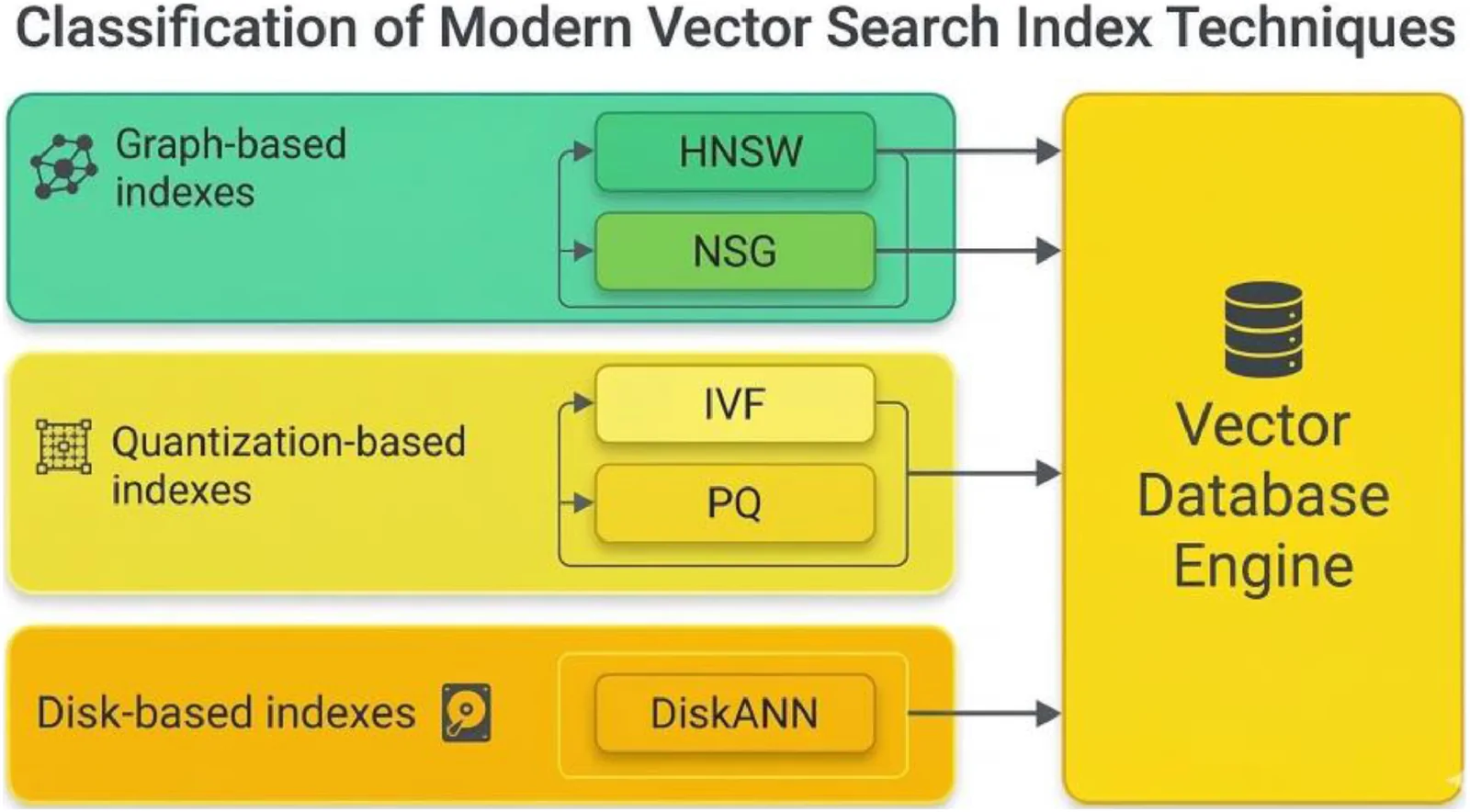
Background view of ANN indexing families, including graph-based, quantization-based, and disk oriented approaches.

### Multi-vector database and workload representation

Each data entry consists of many vector columns whereas a query from the workload can access only some of these vector columns. The probability of queries denotes the access pattern frequency and therefore affects the choice of configuration. The system can handle a synthetic workload where each query accesses only a subset of the available vector columns. Each of the randomly generated queries involves each column independently with probability p=0.3. In case no column is included, one column is selected to make sure that each query is valid. The number of columns is restricted to a maximum of three columns in the main pipeline, and a total of 1,000 workload queries are created. Each query retrieves the top k=10 results. The workload entries are assigned uniform probabilities that sum up to one and are stored in data/workload.pkl so that future runs use the same workload.

The estimator training is done on a random sample that contains 1% of the rows from the database. The same sampled row ids are used for all vector columns in order to maintain the correlation between the multiple vector encodings for the same item. The training process uses the first 50 workload entries and checks extended-k values of 100, 200, 300, 400, and 500. The exact top-k results are calculated using brute force and will be used in estimation.

### Candidate index generation

Following the workload driven candidate generation strategy in [1], the present implementation derives candidate indexes from the vector column combinations observed in workload queries. For each query qi, subsets of qi.vid are enumerated whose size is at least max(1, |qi.vid| − DI). With the default value DI = 2, this process retains candidate indexes whose column coverage is sufficiently close to that of the corresponding query. The dimension of each candidate index is the sum of the dimensions of its constituent vector columns. Seed configurations are then generated from combinations of these candidate indexes, with a maximum seed size determined by SE, which is set to 2 by default.

### Estimator training

Estimator training is carried out once for each database using a random sample of rows. In the main pipeline, this sample is set to 1% of the data. For every column, the system builds a small single column HNSW index on the sampled rows. Training queries are then run on these sample indexes, and the optimizer records the cost proxy and recall behavior for different extended-*k* values. The ground truth for this process is generated using brute-force exact search on the same sampled data.

### Cost estimation

For each sampled single-column index, the optimizer fits a linear model

numDist=a·ek+b

These trained models are then used to estimate scan cost during query planning and configuration search. For multi-column indexes, the implementation does not train a separate model for every possible column combination. Rather, it predicts their behavior using the average coefficients obtained from the associated one column models. Currently, the cost training signal is captured using the observed pair (*ek, ek*). Therefore, in this case, the learned cost model takes the value of *ek* as an estimate of the distance computations made, instead of calculating the distance computations made by the HNSW method. Another piece of information given in the documentation is that the linear association is reliable when *ek* ≥ 100.

### Interpretation of the cost estimator

The currently implemented cost function is meant more to serve as a comparative optimization criterion for ranking candidate queries and indexes rather than a predictive cost metric for wall clock time. Currently, the extended-k parameter is chosen as a substitute for the number of distance computations since no information about the internal HNSW graph traversal counts is available within the optimization process.

Benchmarking serves as an indirect tool for assessing the validity of the selected optimization objective compared to serving performance. One can see from the results that better retrieval does not necessarily imply better serving in terms of lower latency due to the fact that the actual latency depends on the traversal itself, size of the candidate set, memory accesses, dimensions of the index and reranking overhead. Thus, the current study does not imply direct equivalence between the estimated cost and the measured latency.

### Recall estimation

Recall is estimated from sample-based execution traces using a logarithmic model

recall=a·log(ek)+b

Recall estimator is estimated from sample-based traces of execution to understand how extended-k is related to recall quality in training. Recall of single column indexes is modeled using logarithm function to fit the sampled points, while the multi column behavior is estimated from its corresponding column level data. Yet in this current implementation, the trained recall model is not used as the feasibility test in query plan evaluation. Rather, the plan evaluator estimates feasibility of each candidate query plan by retrieving results using temporary sample indexes and then computing the overlap between the sample-based result set and brute force ground truth result set. Thus, it is preferred to use the observed recall of samples to estimate if a query plan meets the recall threshold. The trained recall estimator can thus be considered as part of characterization of the implementation but not a deciding factor in the plan evaluator.

### Query planning

Following the query planning structure in [1], the current implementation decides on the relevance of each index per query and candidate configuration, as well as what extended k-values will be given to each index. The final QueryPlan object will store the query, the extended-k mapping, the cost estimate, and the sample based recall. .

In cases where the number of relevant indexes fulfills the condition |*X*| ≤ 3, the planner enumerates all the possibilities of the relevant extended-*k*. This is because the relevant extended-*k* is derived using rank positions of the ground-truth items. As such, since there are only a few indexes, the use of exhaustive search to come up with the answer is feasible. If |*X*| *>* 3, on the other hand, then the planner will not be able to enumerate all the possibilities; hence, the adoption of an approximation approach, which will rely on sampling of the ground-truth set. For instance, in the repository, the parameter *K_P_ RIME* = 5 means that there will be no more than 2^5^ = 32 subsets.

### Configuration search

Following the configuration search approach in [1], the present implementation employs beam search to come up with the proper index configuration. The seed configurations are tested using query planner, cost estimator, storage validation, and recall evaluator. The top configurations are kept in the beam, and are expanded through the addition of potential indexes, which may later be pruned off. This goes on until there is no further expansion that can be made or when the relative gain drops below the stopping criterion of IM=0.05. Storage validation and caching.

The storage constraint is modeled as a limit on the number of selected indexes:

$$\left|X\right|\leq \theta_{storage}$$

As storage requirements remain constant for HNSW indexes with fixed datasets and maximum degrees, the algorithm considers them equal at present. In terms of speeding up the beam search process, the advantage lies in the reuse of previous decision results, with query IDs combined with certain indexes retrieving their pre-planned decisions through a local cache. On the same token, when searching for (vid*, ek*) within the same planning process, computation is bypassed using temporary memory.

### Final HNSW index construction

The process of searching does not involve the creation of indexes on a large scale for all potential configurations that get evaluated during the process. Rather, it is done once the optimum configuration *X*^∗^ is selected from among all those being evaluated. Once an optimum configuration is determined, the algorithm takes all vectors of each index chosen and creates full scale indexes using hnswlib. The metric used is cosine similarity, and the indexes created are stored under data/indexes/. This clear separation between optimization time estimation and post selection index construction helps avoid unnecessary full-scale indexing work for configurations that are only being considered temporarily.

### Serving-time execution

During serving, the precomputed plan is referred to in order to determine which indexes are to be searched through and which *ek* should be used for each index. Finally, the output of the index scans is joined through union and reranked according to the overall cosine similarity between the candidate documents and the query documents. In doing so, the serving algorithm adopts the same approach followed by the offline cost estimation algorithm, whereby the index scans first yield the candidate set, and then the reranking of the entire query space yields the top-*k* answers.

## Algorithmic implementation of the MINT workflow

The following procedures describe the repository implementation of the principal algorithmic stages of the MINT framework in [1]. They are implementation-oriented pseudocode descriptions of the present codebase and should not be interpreted as newly proposed algorithms. The following procedures restate the verified repository behavior in pseudocode oriented form.

### Procedure 1: Candidate generation and seed construction

1. For each workload query *q*, sort the columns in vid(*q*).
2. Set the minimum subset size to max(1*,* |vid(*q*)| − *DI*).
3. Enumerate all subsets whose size ranges from that minimum to |vid(*q*)|.
4. Create one candidate index per subset, with dimension equal to the sum of covered column dimensions.
5. Generate seed configurations by taking combinations of these candidates of size 1 through min(*SE,* #candidates).
6. Union and deduplicate candidates and seed configurations across all queries.

### Procedure 2: Query planner dispatcher

1. Given query *q* and configuration *X*, keep only indexes whose column sets are subsets of vid(*q*).
2. Build one temporary sample HNSW index per relevant index by concatenating the sampled columns in sorted order.
3. If the number of relevant indexes is at most 3, call Algorithm 1.
4. Otherwise, call Algorithm 2.
5. Return the cheapest plan found, or a fallback plan with *ek* = *k* for all relevant indexes if no plan satisfies the threshold during the search.

### Procedure 3: Algorithm 1 for small configurations

1. For each relevant index, retrieve the rank positions of ground-truth items in the temporary sample index.
2. Construct the set of relevant *ek* values as {0} ∪ {rank + 1}.
3. If there is only one index, evaluate each relevant *ek* directly.
4. If there are multiple indexes, enumerate combinations for all but the last index and search the last index for the cheapest *ek* meeting the recall threshold.
5. For each full assignment, compute scan cost, reranking cost, and recall from the union of retrieved sample items.
6. Return the minimum-cost plan that satisfies the recall threshold.

### Procedure 4: Algorithm 2 for larger configurations

1. Sample up to *K PRIME* items from the query’s ground-truth set using a fixed random seed.
2. Enumerate all subsets of the sampled ground-truth items.
3. Use dynamic programming over indexes and covered subsets, where each state stores the minimum cost and the corresponding *ek* assignments.
4. For each index and each subset, compute the smallest *ek* that covers the desired ground-truth items in the sample ranking.
5. Select the lowest-cost state whose covered fraction meets the recall threshold.
6. Add reranking cost and compute actual recall from sample retrieval overlap before returning the plan.

### Procedure 5: Beam search over configurations

1. Normalize and deduplicate seeds and candidates.
2. Evaluate each seed configuration for storage validity, query coverage, query plan recall, and weighted workload cost.
3. Keep the best configurations up to beam width *BEAM WIDTH*.
4. Expand each beam configuration by adding one new candidate index.
5. Prune globally irrelevant indexes from each expansion.
6. Reevaluate the expanded configurations and keep the best beam again.
7. Stop when there are no expansions or when the improvement falls below *IM* .
8. Return the best valid configuration, or the best-scored fallback if no valid one exists.

### Pipeline stages

The end-to-end pipeline consists of the following stages:

1. Load the multi-vector database and workload.
2. Train cost and recall estimators on a database sample.
3. Generate candidate indexes from workload queries.
4. Generate seed configurations and evaluate them.
5. Run beam search with query planning, caching, and storage validation.
6. Select the best valid configuration.
7. Build full scale HNSW indexes only for the selected configuration.
8. Execute workload queries using cached query plans, index scans, candidate union, and full score reranking.

### Implementation stack

The solution itself has been written in Python 3.11+ with selected libraries for various stages of the workflow. For vectors manipulation, the NumPy library was selected; for constructing and searching HNSW index, the hnswlib library; for estimating the models, scikit-learn; for combinatorics and helper subset operations SciPy; and h5py for data input and output. The requirements.txt file from the repository contains numpy, h5py, hnswlib, scikit-learn, and SciPy. The shared data structures are implemented using Python’s standard library data classes module.

### Module organization

The structure is designed in a way that every key functionality is implemented separately. For example, main.py controls the whole process from data loading and optimization until building the index. The file called config.py contains some crucial variables such as the sampling rate, recall requirement, storage limit, beam search parameters, and initial settings of HNSW. The core of the project is implemented in the following files from the directory src/data: Index, Query, Configuration, WorkloadEntry, and QueryPlan. The code related to estimators is located in the directory src/estimators/, query planning code is in the directory src/planner/, configuration search code in the directory src/searcher/, storage validation code in the directory src/storage/, and the construction code in the directory src/index builder/.

### Query execution model

Once tuned, cached query plans determine the execution of tasks. Not all indices have to be considered; only those with a nonzero value for extended-*k* do. Candidate identifiers are combined using the merging operation, following which sorting takes place according to the total score of each column combination. The top-*k* candidates with the highest scores form the final result set.

### Main pipeline versus benchmark pipeline

There are two execution paths in the repository that have relations to each other but are not identical. First of all, there is an optimizer pipeline located in main.py. In this execution path, the hyperparameters are set as follows: *K* = 10, training takes place on a 1% data sample, while the generated workloads involve queries which are limited by the number of three columns. Another execution path is provided by the benchmark pipeline implemented in benchmark/run benchmark.py and involves such queries that their probability equals *p* = 0*.*5, while *K* = 100. What is also important is that the benchmark employs the 5% data sample and serves it with an additional serving configuration based on the joint indexes for each query type. This distinction is important when interpreting the results section, since benchmark conclusions should be understood in the context of the benchmark setup and not automatically assumed to represent the exact behavior of main.py.

The principal implementation modules and their respective responsibilities are summarized in Table 3.

**Table 3:** Implementation modules and responsibilities.

Module	File(s)	Responsibility	Inputs / Outputs
Entry pipeline	main.py, demo.py	Coordinates the optimizer workflow and demo execution.	Inputs: configuration, datasets, workload. Outputs: selected configuration, built indexes, serving demonstrations.
Configuration constants	config.py	Stores defaults for *DI, SE, IM*, sampling, recall thresholds, storage paths, HNSW settings, and datasets.	Inputs: none. Outputs: constants imported across the repository.
Data models and loading	models.py, loader.py	Defines shared data classes and loads vector columns and workload objects.	Inputs: file paths, formats, dimensions. Outputs: col data, workload entries.
Estimator layer	trainer.py	Samples the database, builds small HNSW indexes, and fits cost and recall models.	Inputs: sampled columns, workload subset. Outputs: trained estimator objects.
Query planner	query planner.py	Selects relevant indexes and extended-*k* values for a query under one configuration.	Inputs: query, configuration, estimators, sample data, ground truth. Output: QueryPlan.
Configuration searcher	candidate generation beam search.py	tGoern.eprya, tes candidates and seeds, evaluates configurations, and runs cached beam search.	Inputs: workload, candidates, seeds, estimators, sample data. Output: selected configuration.
Storage estimator	estimator.py	Checks the cardinality-based storage constraint.	Input: configuration. Output: index count and validity flag.
Index builder	builder.py	Builds persisted HNSW indexes from concatenated vectors and stores metadata.	Inputs: column matrices, configuration, query vector. Outputs: index files, metadata, nearest-neighbor results.
Benchmark pipeline	run benchmark.py	Compares PerColumn serving with benchmark-specific MINT-style serving and saves results.	Inputs: datasets, benchmark queries. Outputs: JSON, CSV, and plot artifacts under benchmark/results/.

## Data and experimental setup

### Dataset construction

A semi-synthetic multi-column vector database forms the basis of the assessment. From distinct sources, each standard column originates separately. During ingestion, truncation aligns every column to the smallest shared number of rows. Stored within the repository, a benchmark output records 400,000 rows per column for that specific test instance.

The implementation and experimental parameter settings used in the main and benchmark pipelines are summarized in Table 4.

**Table 4:** Implementation and experimental parameter settings used in the main and benchmark pipelines.

Category	Parameter	Main pipeline	Benchmark pipeline	Purpose
Workload	Column inclusion probability	0.30	0.50	Probability that a vector column is included in a generated query
Workload	Number of generated queries	1,000	150	Workload size
Workload	Measured benchmark queries	Not applicable	140	Ten initial queries are used as warm-up and excluded from timing
Workload	Maximum query width	3 columns	No explicit cap	Maximum number of vector columns referenced by a query
Retrieval	Top-(k)	10	100	Number of final neighbours requested
Sampling	Database sample fraction	1%	5%	Fraction of database rows used by the respective evaluation pipeline
Candidate generation	(DI)	2	2	Maximum column-count difference between a query and candidate index
Seed generation	(SE)	2	2	Maximum number of indexes in an initial seed configuration
Beam search	Beam width	5	5	Number of configurations retained per iteration
Beam search	Improvement threshold (IM)	0.05	0.05	Relative objective improvement required to continue
Query planner	(K')	5	5	Number of ground-truth items sampled by the dynamic-programming planner
Recall constraint	(\theta_{\mathrm{recall}})	0.90	0.97	Minimum recall used during optimization/evaluation
Storage constraint	(\theta_{\mathrm{storage}})	6 indexes	18 indexes before benchmark-specific augmentation	Cardinality-based limit on selected indexes
Estimator training	Training workload entries	50	Not used in the same form	Queries used to train the sample-based estimators
Estimator training	Extended-(k) values	100, 200, 300, 400, 500	Benchmark-specific execution	Values used to construct estimator observations
HNSW	Distance measure	Cosine	Cosine	Similarity space
HNSW	Maximum graph degree (M)	16	16	Maximum number of graph connections
HNSW	ef_construction	200	200	Construction-time search width
HNSW	ef_search	(\max(100,ek))	(\max(100,ek))	Query-time search width
Reproducibility	Random seed	42	42	Repeatable sampling and workload generation

**Fig. 6:**
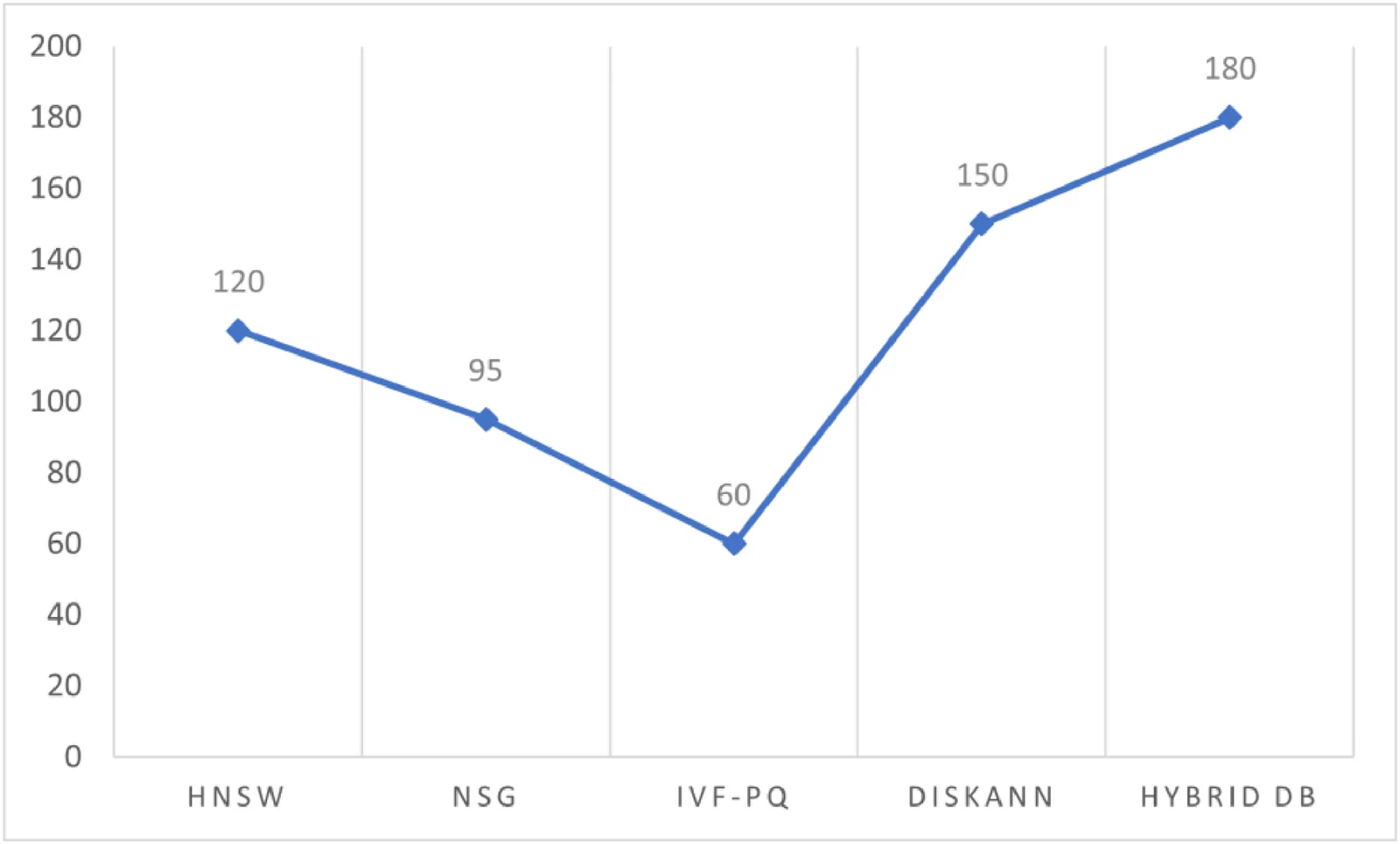
Comparison of index construction time across representative ANN techniques, highlighting the importance of avoiding unnecessary full-scale index builds.

The comparison of index-construction time across representative ANN techniques is shown in Fig. 6, highlighting the motivation for avoiding unnecessary full-scale index construction during configuration search.

Verification details for the standard datasets are summarized in Table 5.

**Table 5:** **.** Default dataset columns declared in the repository.

Col. ID	File	Format	Dim.	Role / Source
0	glove.6B.50d.txt	txt	50	GloVe word embeddings, first vector column
1	glove.6B.100d.txt	txt	100	GloVe word embeddings, second vector column
2	glove.6B.200d.txt	txt	200	GloVe word embeddings, third vector column
3	sift-128-euclidean.hdf5	hdf5	128	SIFT image descriptors
4	deep1M base.fbin	fbin	96	Deep1M image features
5	database music100.bin	bin	100	Music audio embeddings

Although the data loader handles three distinct storage types shown in the source, its behavior changes per format. When processing text files, it reads one line at a time but ignores the initial entry on every row. With HDF5, loading occurs strictly from the train set inside the file. Instead of relying on headers, .fbin and .bin inputs are interpreted directly as flat sequences of float32 values, where the number of rows comes from dividing total size by dimension. On first run, the tool generates the index output folder automatically this prevents future indexing operations from breaking due to an absent directory.

### Workload generation

One setup builds a repeatable synthetic workload saved on disk the other creates unique queries for testing speed and accuracy. These two methods live in the same repo yet serve distinct roles. What runs once stays stored; what runs per test stays fresh.

One random row from every chosen column becomes the query vector in the main pipeline; these form query probability pairs where each holds equal weight. Though it uses the same method for picking columns, the benchmark pipeline skips limits on how many values appear per query. Queries aim to retrieve *k* = 100 items there, with measurements taken only after an initial warmup period. What sets them apart lies in what gets reported planner derived weights guiding the optimizer stay out of the benchmark results.

### Training and optimization settings

The estimator training phase in the main pipeline is based on a 1% random sample of database rows, whereas the saved benchmark artifact reports that a larger 5% sample is used during benchmark execution. The default recall thresholds are documented and implemented as 90% for large datasets and 97% for small datasets. In main.py, beam search is directly called with the large dataset recall threshold of 0*.*90, and the storage threshold is set equal to the number of columns in the database. In the benchmark pipeline, the threshold recorded in the saved artifact is 0*.*97, and the beam search call uses a storage threshold equal to three times the number of columns before the benchmark specific configuration augmentation is applied.

Candidate generation uses *DI* = 2, seed generation uses *SE* = 2, the planner for larger configurations uses *K_P_ RIME* = 5, and beam search stops once the improvement falls below *IM* = 0*.*05. For final HNSW construction, the implementation uses cosine similarity as the distance measure and a maximum graph degree of 16. The index builder initializes HNSW indexes with ef construction = 200, while the query time loading logic sets the search parameter to at least 100 and also ensures that it is at least as large as the requested *ek* value.

The verified workload and query generation settings for the two pipelines are summarized in Table 6.

**Table 6:** Verified workload and query generation settings.

Aspect	Main Optimizer Pipeline	Benchmark Pipeline
Implementation	loader.py	run benchmark.py
Column inclusion probability	*WORKLOAD P* = 0*.*3	*BENCH P* = 0*.*5
Random seed	42	42
Minimum columns per query	At least one, forced if random draw selects none	At least one, forced if random draw selects none
Maximum columns per query	Capped at *MAX QUERY COLS* = 3	No explicit cap
Top-*k* value	*K* = 10	*BENCH K* = 100
Number of queries	*NUM QUERIES* = 1000	*TOTAL QUERIES* = 150
Warmup usage	Not applicable in the generator	First 10 queries discarded before timing
Probability assignment	Uniform probabilities summing to 1 across the workload	Benchmark query list is used for measured evaluation, not as a normalized workload objective

### Experimental environment

Implementation relies on CPU-based Python and supports Python 3.11 and above, along with NumPy, hnswlib, scikit learn, SciPy, and h5py libraries. Dependency information needed to replicate the software environment is listed in the public repository via requirements.txt file. HNSW graph generation employs cosine distance metric, M=16 and ef_construction = 200. During query time, the search parameter is configured to at least 100 and increases when necessary based on requested extended-k.

The archived benchmark artifact lacks any machine-specific metadata such as specific CPU, amount of RAM, operating system version, and the precise snapshot of package versions used for historical execution. Therefore, it is impossible to reliably recover this information and infer it retroactively. The mentioned restriction is clearly stated here to make sure that timing benchmarks are treated as measurements done within the execution environment from archive and not as hardware independent ones.

### Experimental protocol

The verified experimental protocol and corresponding outputs are summarized in Table 7.

**Table 7:** **.** Verified experimental protocol and outputs.

Stage	Implementation	Key Verified Settings	Output
Database loading	loader.py	Load declared dataset files, truncate all columns to common row count, create index output directory	In-memory column matrices
Main workload generation	loader.py	*p* = 0*.*3, cap width at 3 columns, 1000 queries, uniform probabilities, persistent cache path	Cached workload file and workload objects
Estimator training	trainer.py	Sample fraction 1% in main pipeline, first 50 workload entries, training *ek* ∈ {100*,* 200*,* 300*,* 400*,* 500}	Cost and recall estimator objects
Main configuration search	main.py + searcher	Per-column seed added explicitly, *θ*recall = 0*.*90, *θ*storage = 6 for six columns	Selected configuration *X*∗
Index building	builder.py	Full concatenated vectors, cosine HNSW, *M* = 16, persisted binary index files and metadata	Built index files under data/indexes/
Benchmark query generation	run benchmark.py	*p* = 0*.*5, 150 total queries, 10 warmup queries, 140 measured queries, *k* = 100	Query list grouped by type
Benchmark serving comparison	run benchmark.py	Compare PerColumn search with benchmark-specific MINT-style joint-index serving on a 5% sample	JSON, CSV, and PNG result artifacts

### Evaluation metrics

The repository supports two categories of evaluation metrics.

1. *Optimizer-internal cost proxy*: Used during configuration search as a weighted sum of estimated scan cost and reranking cost.
2. *Artifact-backed benchmark metrics*: Measured latency in milliseconds, recall at the benchmark’s top-*k*, pass/fail status relative to the applied threshold, query type counts, and the saved benchmark configuration.

These metrics should be interpreted separately. Estimated cost is used to rank configurations during search, whereas the benchmark artifact records wall clock serving behavior under a benchmark-specific execution setup.

## Ablation and parameter sensitivity plan

Right now, the version of the repo lacks full tests that remove parts of the optimizer’s main controls. Even so, the way the code is built makes it possible to design careful experiments later. Aiming to adjust just one setting at once, keeping data, starting conditions, and testing steps unchanged, researchers could track shifts in search volume, planning load, space needed, setup performance, predicted expense, retrieval success, and actual runtime patterns.

**Fig. 7:**
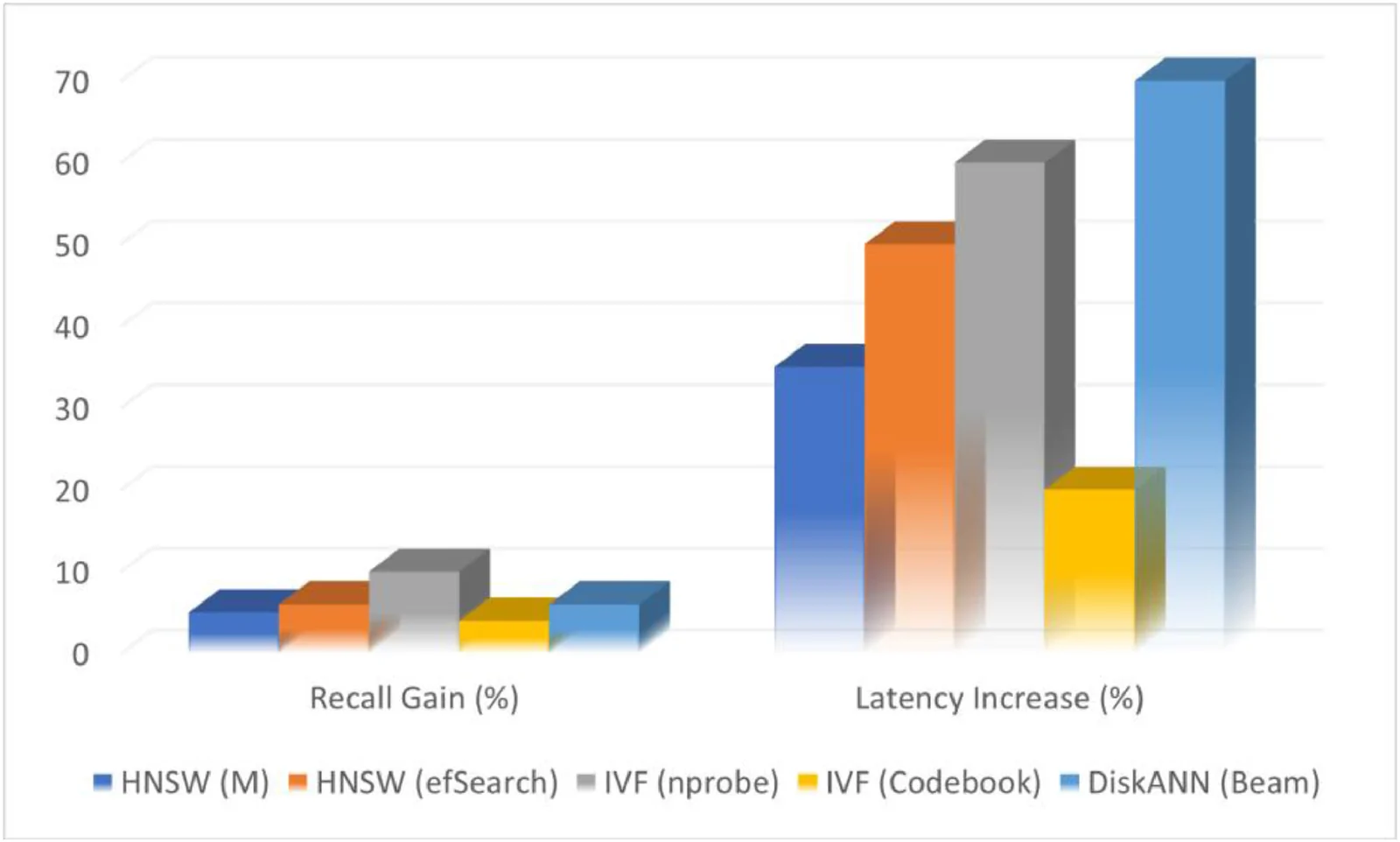
Impact of key ANN parameters on recall improvement and latency overhead.

A logical starting point involves locking down the testing framework pick either the primary optimization setup or the evaluation-only version, never blend both when isolating variables. Following that, select a control configuration matching what the codebase uses by default. Then explore slight adjustments across just one setting at a time, keeping randomness and data inputs unchanged throughout each trial. Among recorded details, include figures like candidate volume, seeds used, beam-search rounds, adjusted workload expense, plus how many indexes were picked. Evaluation should cover outcomes too recall rates and response delays under test conditions. These observations make it possible to spot adjustments that genuinely lift performance, separate from those inflating effort with little real gain. What shows up here shapes understanding of trade-offs. The expected impact of key ANN parameters on recall improvement and latency overhead is illustrated in Fig. 7.

The potential qualitative effect of each of the principal parameters can be gleaned through a verified implementation. The effect of the parameters *DI, SE*, and *BEAM_W_ IDTH* is a direct influence on the portion of the search space that will be searched when creating candidates and conducting the beam search. The parameters *K_P_ RIME* and *SAMPLE_F_ RAC* contribute to estimator accuracy and the quality of approximation provided by the planner; however, they also contribute to increased planning overhead. Constraint parameters *θ*_recall_ and *θ*_storage_ determine how hard the optimizer may try to optimize the plan for better cost efficiency or lower index selection at the expense of plan quality. Workload-generation parameters like *K, WORKLOAD_P_,* and *MAX_Q_UERY_C_OLS* affect the structure of the generated workloads and therefore have an indirect effect on candidate diversity and utility of multicol indexing.

Consideration of both angles provides a clear insight while analyzing entirely isolated elements. From an optimization perspective, note how many candidates come out, number of seeds utilized, overall number of setups analyzed, time consumed during search process, setup size after optimization and the modified workload cost as well. While analyzing server behavior, evaluate retrieval efficiency, latency and space requirements following the same protocol every time. Distinguishing between these perspectives is important because some parameters affect only preparatory operations, while others determine performance results.

## Method validation

The updated validation tests the entire optimization process chain consisting of workload generation, estimator training, candidate generation, beam search configuration selection, HNSW build and query execution at serving time. The primary difference between the new validation test and the old benchmarking artifact lies in the fact that the new validation test does not augment the beam search output with additional joint indexes for observed queries as in the old benchmarking artifact and directly evaluates the configuration chosen by beam search itself without any augmentation.

All necessary information including the chosen configuration, objective of the optimization, the runtimes of configuration search and index builds, measurements of recall and latency on query level, software and hardware environment for the revised experiments have been recorded. Unless stated otherwise, all comparisons in this section compare the beam-search configuration without any augmentation.

### Selected index configuration

The optimizer returns the best valid configuration *X*^∗^ under the workload, recall, and storage constraints. For the main pipeline in main.py, the exact persisted *X*^∗^ is not present in the repository snapshot and therefore remains: The repository snapshot does not persist the selected main.py configuration *X*^∗^. Therefore, the saved benchmark configuration is reported separately from the unavailable main pipeline output.

In addition to this, the repository includes an older benchmark artifact where the configuration generated by the search algorithm was further extended by a joint index for each unique query type in the benchmark dataset, totaling 57 indexes. Since this happens post-configuration search, the configuration generated in this manner is not considered the output of MINT beam-search optimizer and is not used as proof of the performance of the tuner. The data is kept simply for demonstrating how an extensively augmented joint index configuration behaves.

### Workload cost comparison

The primary optimization objective is the probability weighted average workload cost estimated by the planner and configuration searcher. The repository snapshot does not contain a persisted value for this objective from the main pipeline. Therefore, the repository snapshot does not contain a persisted optimized weighted workload cost from main.py. Ther The same applies to a strict baseline comparison on the optimizer’s internal objective.

The benchmark artifact records measured latency and recall under a separate benchmark protocol, but it does not provide the planner’s weighted workload objective. Consequently, any direct cost comparison in this subsection must remain run dependent unless the main pipeline is executed and its outputs are saved.

The planned ablation dimensions, their expected effects, and the corresponding evaluation measures are summarized in Table 8.

**Table 8:** **.** Planned ablation dimensions for optimizer sensitivity analysis.

Parameter	Role	Expected Influence	Metric to Observe
*DI*	Maximum query-to-index size gap during candidate generation and relevance filtering	Larger values increase candidate diversity and search-space size, may improve configuration quality for wider queries, but can increase planning and search cost	Candidate count, relevant-index count, beam-search time, recall, selected index count
*SE*	Maximum number of indexes per seed configuration	Larger values create richer starting points for beam search but increase seed count and seed-evaluation cost	Seed count, seed-evaluation time, best-seed cost, final configuration cost
*IM*	Improvement threshold for stopping beam search	Smaller values encourage longer search and potentially better configurations, while larger values stop earlier with lower optimization overhead	Beam iterations, total search time, final weighted cost, selected configuration size
*BEAM WID*	*T* Number of configurations retained per search layer	Larger widths explore more of the configuration space and may improve robustness, but increase evaluation cost substantially	Configurations evaluated, search time, final weighted cost, recall validity rate
*K PRIME*	Ground-truth sample size in the DP planner	Larger values improve DP coverage fidelity for large configurations but increase planner state count and per-query planning cost	Planner runtime, number of DP states, plan recall, weighted workload cost
*SAMPLE FR*	Fraction of rows used for estimator training or sample-based evaluation	Larger samples can improve estimator fidelity and sample-based ground truth quality, but increase preprocessing, memory use, and planning time	Estimator training time, sample size, plan recall stability, benchmark latency variance
*θ*recall	Minimum recall constraint	Higher thresholds force more conservative plans and possibly larger or more expensive configurations; lower thresholds allow cheaper but potentially less accurate plans	Plan recall, weighted cost, selected index count, pass/fail rate
*θ*storage	Maximum allowed number of indexes	Larger values expand the feasible configuration space and can improve recall or lower cost, but may increase storage usage; smaller values force more aggressive compression of the design	Selected index count, configuration validity, weighted cost, recall
*K*	Final top-*k* retrieval target in the main pipeline	Larger values increase reranking cost and may enlarge the set of meaningful *ek* values, affecting plan complexity and evaluation effort	Plan cost, reranking cost, planner runtime, recall@k
*WORKLOAD*	*P*er-column inclusion probability in the main synthetic workload generator	Larger values produce wider queries and encourage more multi-column candidates, increasing search complexity and the potential value of joint indexes	Query-width distribution, candidate count, selected multi-column indexes, recall, latency
*MAX QUER*	COLS query width in the main workload generator	Larger caps allow more complex queries and broader candidate spaces, while smaller caps simplify planning and preserve single-column relevance	Query-width distribution, relevant-index count, weighted cost, selected index structure

### Recall and latency observations from saved benchmark artifacts

The repository indeed has the values of recall and latency for the pipeline under benchmarking. These are summarized in Table 9 according to the overall data, while Table 10 shows them by query type.

**Table 9:** **.** Overall saved benchmark results.

Metric	Value
PerColumn average latency	2.9175 ms
MINT-style average latency	8.4114 ms
Reported speedup ratio	0.347
PerColumn average recall	0.3438
MINT-style average recall	0.9666
Applied recall threshold	0.97
Measured queries	140

**Table 10:** Saved benchmark results by query type.

Type	Queries	PerCol ms	MINT ms	Speedup	PerCol Recall	MINT Recall	PerCol Status	MINT Status
1-col	14	0.6237	2.6486	0.235	0.9693	0.9700	FAIL (need 97%)	PASS
2-col	39	1.6574	7.8225	0.212	0.3959	0.9508	FAIL (need 97%)	FAIL (need 97%)
3-col	45	3.5866	7.9937	0.449	0.2529	0.9684	FAIL (need 97%)	FAIL (need 97%)
4-col	28	4.1785	12.3773	0.338	0.1989	0.9779	FAIL (need 97%)	PASS
5-col	11	4.0355	9.6601	0.418	0.1609	0.9755	FAIL (need 97%)	PASS
6-col	3	4.0997	7.6308	0.537	0.1333	0.9933	FAIL (need 97%)	PASS

These benchmarks give rise to three primary conclusions. Firstly, the MINT-based approach of benchmarking is considerably more efficient in terms of the recall compared to the PerColumn approach, especially when considering multi-column queries. Secondly, the improved recall level is followed by a higher latency of the query processing, and, hence, the benchmark fails to deliver any actual wall clock speedup. Thirdly, the recall value depends on the query width with one-column, four-column, five-column, and six-column queries reaching or surpassing the 0.97 threshold.

**Fig. 8:**
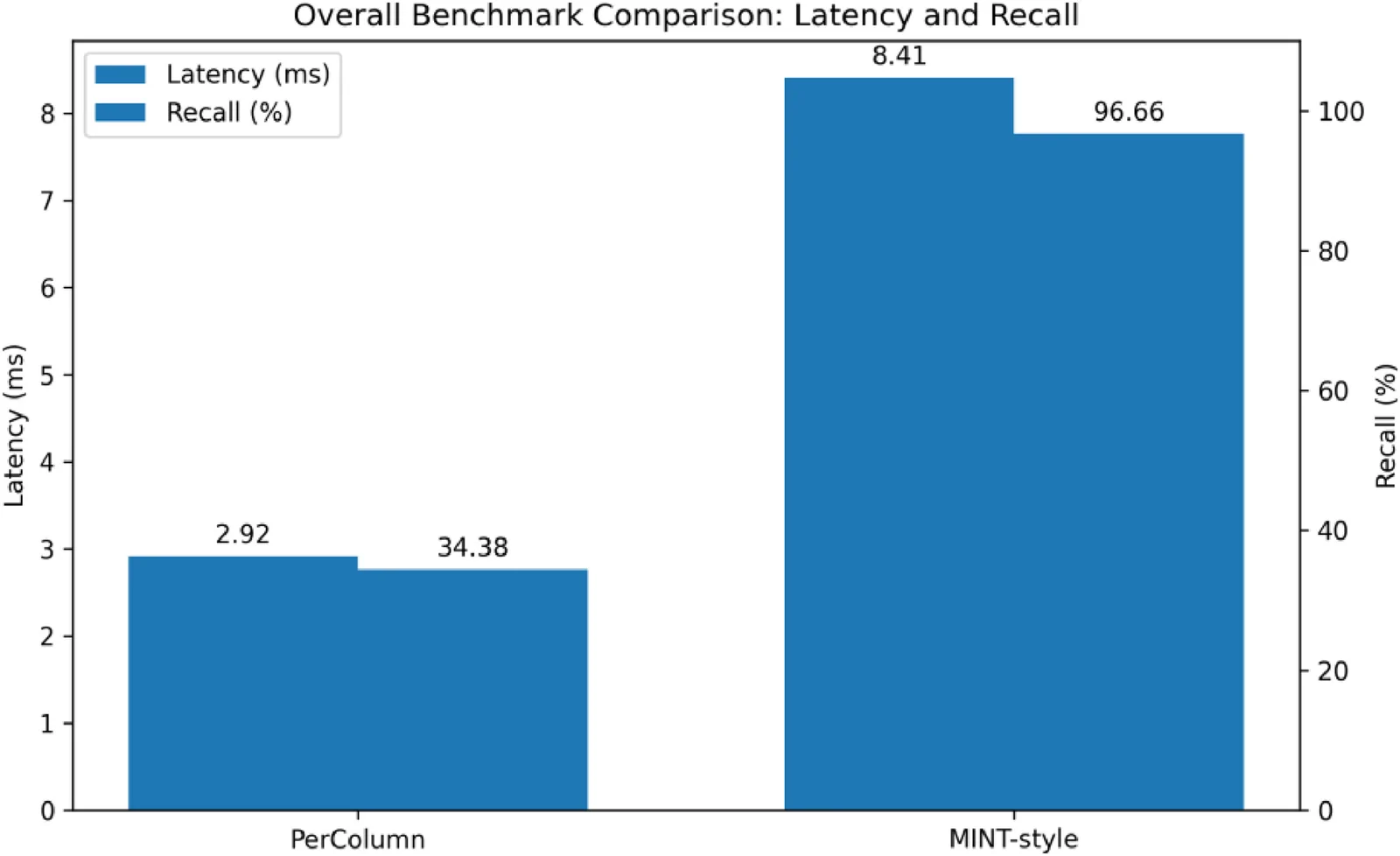
Overall benchmark comparison between PerColumn and MINT-style serving in terms of average latency and recall.

**Fig. 9:**
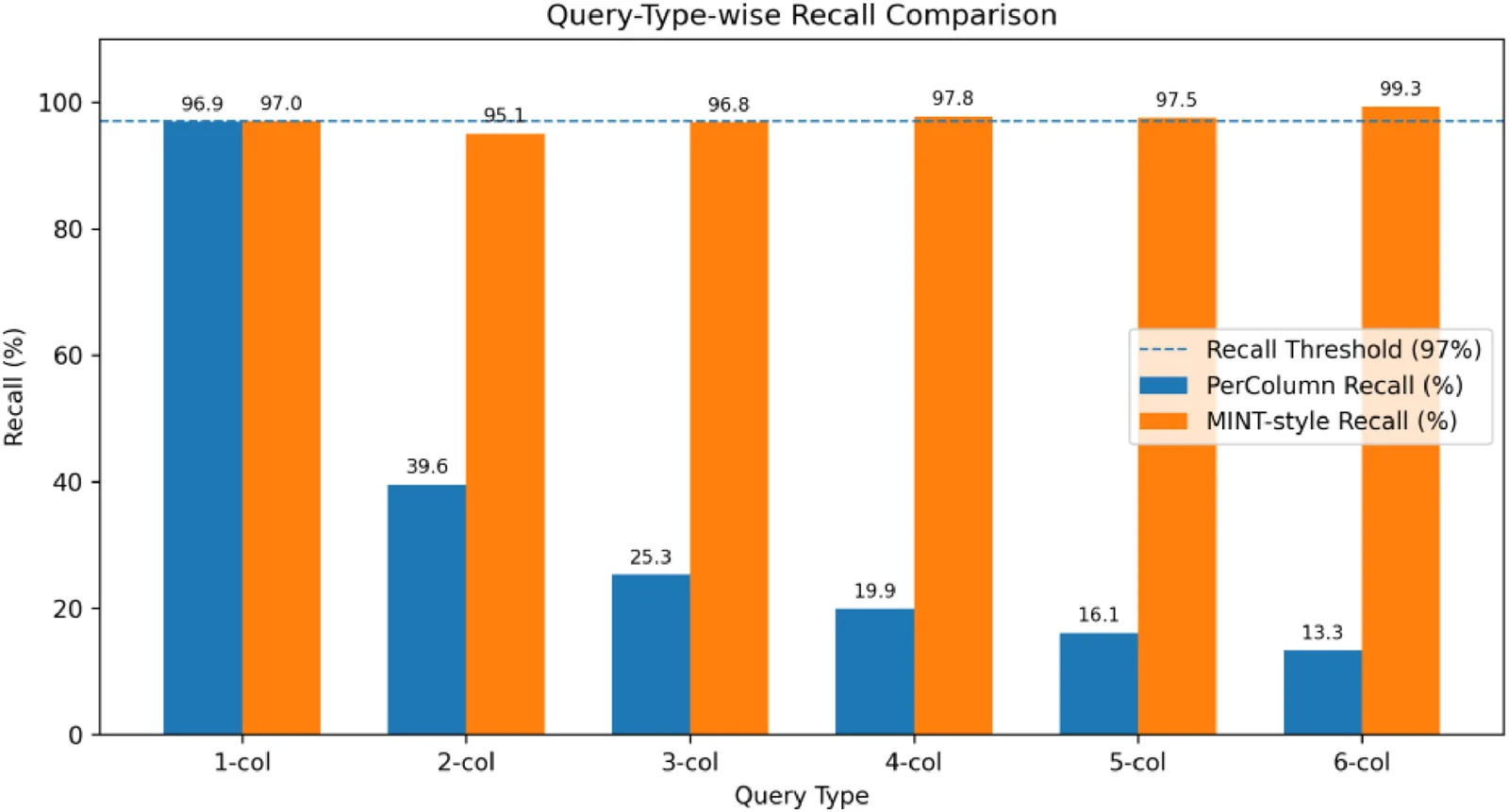
Query-type-wise recall comparison between PerColumn and MINT-style serving across one-column to six-column query workloads.

To visually summarize the saved benchmark results, Fig. 8 compares the overall latency and recall values of PerColumn and MINT-style serving. Fig. 9 further shows recall behavior across different query widths, indicating that the MINT-style serving approach provides substantially higher recall for multi-column query workloads.

### Storage constraint validation

Space limits during setup depend on how many indexes are picked, not on memory size. In main.py, the limit matches the column count set to 6 for the standard six column dataset. During testing, beam search runs with a cutoff set at triple that number: 18. Still, once expanded for evaluation purposes, the stored test setup includes 57 indexes in total. So, the benchmark artifact ought to avoid supporting claims about meeting the initial beamsearch storage limit in production settings. Instead, the following distinction should be maintained:

• Main pipeline storage budget in code: |*X*| ≤ 6.
• Benchmark beam-search budget in code before augmentation: |*X*| ≤ 18.
• Saved benchmark final configuration size after augmentation: 57 indexes.

The exact selected configuration size for a persisted main.py run is not available in the current repository snapshot.

### Index construction and execution observations

After selecting *X*^∗^, the implementation builds full scale HNSW indexes only for the final chosen configuration. This can be seen directly from the builder logic and the structure of the main pipeline. Nevertheless, the current version of the code repository does not have any saved information on the build time, as well as an accurate full size logging data collected at query time using main.py.

The repository snapshot does not include persisted measurements for full-scale index build time, full scale query execution time from main.py, or additional controlled run observations. These missing measurements are therefore reported as reproducibility and evaluation limitations rather than estimated.

The benchmark pipeline, however, does include measured serving times in a sample-based setup and reports 140 measured queries after removing a 10 query warmup. The saved query type breakdown contains 15 one column queries, 41 two-column queries, 51 three-column queries, 28 four-column queries, 12 five-column queries, and 3 six-column queries before warmup removal. This artifact shows that the benchmark evaluates a heterogeneous multi-vector workload, rather than testing only one fixed query shape.

The new evaluation separates the behavior of the optimized beam search selected configuration from the previous benchmark specific augmented configuration. The difference is needed because the addition of joint indexes to cover all the possible queries types can be very beneficial for retrieval coverage without showing that the optimizer actually chose them.

Thus, the main concern of the experiments conducted in this work is whether the workload aware configuration search provides any useful configuration in comparison with other baselines under the constraints of fixed recall and storage capacity. The improvements in recall, estimated optimization cost, and measured latency are evaluated separately since these metrics are not equivalent. Specifically, an improvement in recall does not mean the decrease in measured query latency and vice versa, while the cost optimization in the optimizer does not mean its speedup.

The implementation also demonstrates an important discrepancy between the goals of the algorithmic optimization and the serving behavior of the ANN. The configuration search uses sample-based planning and cost estimation based on this samples, while the serving time is dependent on HNSW traversal, candidate set size, reranking, hardware, and workload specifics. Thus, the results are restricted to the environments tested in the revised experiments.

## Limitations

### Applicability and situations with reduced benefit

Workload-aware parameter tuning can have the largest impact when there are multiple vector fields in the database and the workload involves accessing different subsets of the vector fields. Other cases where load aware tuning is less useful include when queries use mostly one vector field, the query distribution is roughly uniform, or there is a previously built index which meets all the recall and latency requirements. The off-line process of estimating and searching for parameters might not be justified for smaller databases and seldom used applications.

The approach makes the assumption that the workload which was used for optimizing is representative of the future queries. In case there is a drastic change in the query distribution, the vector fields used, or the recall requirements, the configuration chosen will no longer be optimal. Currently there is no online workload adaptation mechanism.

There may also be limits to practical utility in light of memory limitations on a byte for byte basis, in the sense that the current memory model is based on the number of selected indices, and not their memory or disk size. Likewise, the internal cost model approximates the computation cost for distance calculation and may not order results in the same way as latencies would. It follows that the current algorithm is most readily suited to static CPU HNSW search.

### Implementation limitations

Several limitations follow directly from the current codebase.

• The current cost estimator does not directly measure the actual internal distance computations performed by HNSW. Instead, during training, it stores (*ek, ek*) pairs and treats *ek* itself as a proxy value for numDist.
• Although the recall estimator is trained as part of the implementation, the planner does not use the trained recall model inside the final plan evaluation loop. Instead, it computes recall by checking the actual overlap between retrieved results and ground-truth results on the sampled data.
• The storage estimator is still quite simple because it only counts the number of indexes selected. It does not yet estimate byte level storage, memory usage, or the build time footprint of the created indexes.
• The new experiments retain the same beam search settings, optimization goals, timing measurements, and experimental environment used. Yet, the results continue to be dependent on the structure of the dataset used, the workload distribution, the HNSW implementation, and the hardware environment. More experiments on bigger datasets and other families of ANN indexes need to be conducted to prove generalizability.

### Threats to internal validity

The benchmark artifact and the main optimizer pipeline are connected, but they do not evaluate the system under exactly the same conditions. In the main pipeline, workload generation uses *p* = 0*.*3, *K* = 10, and limits query width to at most three columns. In contrast, the benchmark uses *p* = 0*.*5, *K* = 100, does not apply a query width cap, and follows a separate serving model. Therefore, the benchmark results should be interpreted as evidence for that specific benchmark setting, rather than being directly generalized to the exact optimization setup used in main.py.

### Threats to construct validity

Because the optimizer aims at a predicted cost instead of real world speed, its choices might not match observed performance. A design decision drives this approach yet results on timing tests can still differ from what the system ranks internally. In much the same way, how storage is modeled now counts indexes instead of tracking exact byte usage. That leads the storage limit to reflect only a rough idea of resource allocation.

### Threats to external validity

A six-column setup forms the standard data collection, created partly from real sources like GloVe, SIFT, Deep1M, and Music100. While helpful for testing how systems run, this structure misses elements found in live environments changes over time, searches using extra attributes, combined vector and relational queries, or massive networked lookups. Testing performance relies on snapshots instead of complete stored indexes processed through consistent machine configurations.

### Reproducibility considerations

Although the snapshot includes code and default settings along with performance records, it lacks stored raw data, device details, or a saved execution history of main.py. To replicate results exactly, one must reload the specified dataset locations before launching the procedures within an isolated setup.

## Data and code availability

The source code used in this work is openly accessible at https://github.com/1Ninad/vector-index-optimizer. The repository holds the source code of the implementation, configuration parameters, workload generation tools, estimator training, query planner, beam search configuration optimization, HNSW index building, serving system, benchmarking tools, and testing.

The datasets used for experiments are not distributed via the repository due to their size and being hosted elsewhere. The configurations and the file structure of the datasets GloVe, SIFT, Deep1M, and Music100 are provided by the repository. Benchmarking results and artifacts of the experiments done are included in the repository wherever possible. The historical main pipeline output that was not saved is noted in the manuscript as reproducibility restrictions.

## Ethics statements

None.

## CRediT authorship contribution statement

**Sharda Yashwant Salunkhe:** Supervision, Validation, Resources, Writing – review & editing. **Kuldeep Vayadande:** Conceptualization, Methodology, Supervision, Formal analysis, Writing – review & editing. **Hridaynath Pandurang Khandagale:** Supervision, Validation, Resources, Writing – review & editing. **Pavitha Nooji:** Supervision, Validation, Resources, Writing – review & editing. **Mangesh Hajare:** Supervision, Validation, Resources, Writing – review & editing. **Krishna Prakasha:** Supervision, Validation, Resources, Writing – review & editing. **Rakhi Bhardwaj:** Methodology, Software, Validation, Formal analysis, Investigation, Data curation, Visualization, Writing – original draft. **Manasi Phand:** Methodology, Software, Validation, Formal analysis, Investigation, Data curation, Visualization, Writing – original draft. **Anish Katariya:** Methodology, Software, Validation, Formal analysis, Investigation, Data curation, Visualization, Writing – original draft. **Ninad Kale:** Methodology, Software, Validation, Formal analysis, Investigation, Data curation, Visualization, Writing – original draft. **Amey Kharade:** Methodology, Software, Validation, Formal analysis, Investigation, Data curation, Visualization, Writing – original draft.

## Declaration of competing interest

The authors declare that they have no known competing financial interests or personal relationships that could have appeared to influence the work reported in this paper.

## Data Availability

Data will be made available on request.
