# Supplementary material for: Implementation and experimental evaluation of mint for workload-aware approximate nearest neighbor index tuning in multi-vector databases
Source: MethodsX. 2026 Sep 6;17:104151. doi: 10.1016/j.mex.2026.104151 (PMC13586876; doi:10.1016/j.mex.2026.104151)
Supplement: Supplementary file 1 [file mmc1.pdf]

Date -12/08/2026

To,

Editor,

MethodX Journal

Subject: Authorship Change Request – Submission MEX-D-26-01278R2

Ref.: Submission MEX-D-26-01278R2
Article title: *Implementation and Experimental Evaluation of MINT for Workload-Aware Approximate Nearest Neighbor Index Tuning in Multi-Vector Databases*
Journal Name: *MethodsX*

Thank you for your communication regarding the authorship change in our revised submission.

We respectfully request your approval for the addition of **Dr. Rakhi Bhardwaj** as a co-author of the revised manuscript entitled:

"Implementation and Experimental Evaluation of MINT for Workload-Aware Approximate Nearest Neighbor Index Tuning in Multi-Vector Databases"

The reason for this authorship change is that **Dr. Rakhi Bhardwaj became substantially involved during the later stage of the manuscript revision, particularly while addressing the comments and suggestions raised by the reviewers.** Her contribution occurred after the original submission and therefore she was not included in the initial author list.

During the revision process, we sought Dr. Rakhi Bhardwaj's assistance and opinion regarding the revisions required in response to the reviewers' comments. She provided valuable input and suggestions that helped us improve and revise the manuscript. She also provided her valuable opinion and assistance regarding the **preparation and presentation of the Graphical Abstract** and other aspects of the revised manuscript.

These contributions were made during the later stage of manuscript preparation and revision and were therefore not part of the original submission. Based on her contributions to the revision and improvement of the manuscript, we respectfully request that she be included as a co-author.

We confirm that **all existing authors have discussed and unanimously agreed to this authorship change**, and **Dr. Rakhi Bhardwaj has also agreed to be added as a co-author**. All authors are willing to provide written confirmation and signatures as required by Elsevier.

We sincerely apologize for not including Dr. Rakhi Bhardwaj in the original submission and respectfully request the Editor's consideration and approval of this authorship change.

We have completed the **Elsevier Authorship Change Request Form** and attached it with the revised submission as requested.

We further confirm that **no additional authorship changes will be requested** unless specifically required and approved by the Editor.

Thank you very much for your consideration.

Kind regards,

Dr. Krishna Prakasha
School of Computer Engineering
Manipal Institute of Technology, Manipal

Authorship change request

Important information – please read before continuing.

How to use this form

- This form is to be completed by the Corresponding Author to request any change in authorship (additions, removals, or reordering) after the submission of a manuscript, including changes in Corresponding Authors, if any.
- Changes to the author list must not be made in the journal's **Editorial Manager** system without submitting this form. Unauthorized authorship changes will result in the rejection of your submission, or retraction if the article has already been published.
- Authorship changes are not allowed after manuscript acceptance. It is also not possible to submit this form after your manuscript has been rejected, for any reason.
- Do not use this form for name changes or corrections.
- This form consists of three parts, all of which should be completed prior to submission.

A note about author disputes

The publisher and editor cannot investigate or mediate any authorship disputes.

If you are unable to obtain agreement from all authors, including those you intend to remove, we recommend seeking guidance from your institution. We will not consider your change request and will not proceed with the publication of your manuscript until all outstanding authorship disputes are resolved.

Before completing the form

- All authors should carefully review the "Duties of Authors" section of the Elsevier publishing ethics policy, particularly the sections on:
  - Authorship of the paper
  - The use of generative AI and AI-assisted technologies in scientific writing and in figures, images and artwork
- Please also carefully review the submission journal's "Guide for authors" (this might also be referred to as "Instructions for authors"), because some journals may have additional authorship criteria (an example is the ICMJE guidelines for authorship).
- Be prepared to enter the full name, email address, and institution for every author on the manuscript. This information must match the author list in the submission system.

How to submit this form

After completing this form according to the above instructions, it should be submitted through the journal's **Editorial Manager** system with your revised manuscript via the "Submissions Needing Revision" link.

(*continued from the previous page*)

In the “Attach Files” step, use the “Cover letter” item type and in the description, type “Authorship change request form” and upload this form. Make corresponding changes in the Manuscript Data step before completing your revision submission.

For additional guidance, view the tutorial on using Editorial Manager for this process in the Journal Publishing Support Center.

Once submitted, the information in this form will become part of the revision submission record.

Part 1. General submission information

To be completed by **the Corresponding Author**.

Manuscript details

Journal title	MethodX
Manuscript and/ or article number	MEX-D-26-01278R2
Manuscript title	Implementation and Experimental Evaluation of MINT for Workload-Aware Approximate Neares

Change(s) requested (check all that apply)

☑ Add new author(s)	☐ Remove author(s)
☐ Change the Corresponding Author	☐ Change the order of authors*

* If ONLY changing order of existing authors, go directly to Part 3. Do not complete Part 2.

Part 2. Indicate author(s) to be added or removed

For each author to be added or removed complete one template below.**

In the "**Reason for this change**" section, please include as much detail as possible so we can evaluate if the change is approved. At a minimum, this should include explanations for both:

- why the change is being requested, and
- why the author was/was not included in the original author list.

Important Note: If this part is either not provided, incomplete, or the reasons provide insufficient detail or do not address the points above, your request will be denied, and your submission may be rejected.

2.1 Author change information

Given/first name(s)	Dr. Rakhi
Family/last name	Bhardwaj
Email address	rakhi.bhardwaj@vit.edu
Institution	Vishwakarma Institute of Technology, Pune

Change(s) requested for this author ☐ Remove author ☑ Add new author ☐ Make Corresponding Author

Author's Individual contributions (required for author additions only) see CRediT Contributor Roles Taxonomy

☐ Conceptualization	☐ Data curation	☐ Formal analysis
☐ Funding acquisition	☐ Investigation	☐ Methodology
☐ Project administration	☐ Resources	☑ Software
☐ Supervision	☐ Validation	☐ Visualization
☐ Writing – original draft	☑ Writing – review & editing	

Reason for this change

The reason for this authorship change is that Dr. Rakhi Bhardwaj became substantially involved during the later stage of the manuscript revision, particularly while addressing the comments and suggestions raised by the reviewers. Her contribution occurred after the original submission and therefore she was not included in the initial author list.
During the revision process, we sought Dr. Rakhi Bhardwaj's assistance and opinion regarding the revisions required in response to the reviewers' comments. She provided valuable input and suggestions that helped us improve and revise the manuscript. She also provided her valuable opinion and assistance regarding the preparation and presentation of the Graphical Abstract and other aspects of the revised manuscript.
These contributions were made during the later stage of manuscript preparation and revision and were therefore not part of the original submission. Based on her contributions to the revision and improvement of the manuscript, we respectfully request that she be included as a co-author.

2.2 Author change information

Given/first name(s)	
Family/last name	
Email address	@
Institution	

Change(s) requested for this author ☐ Remove author ☐ Add new author ☐ Make Corresponding Author

Author's Individual contributions (required for author additions only)
see CRediT Contributor Roles Taxonomy

☐ Conceptualization	☐ Data curation	☐ Formal analysis
☐ Funding acquisition	☐ Investigation	☐ Methodology
☐ Project administration	☐ Resources	☐ Software
☐ Supervision	☐ Validation	☐ Visualization
☐ Writing – original draft	☐ Writing – review & editing	

Reason for this change

2.3 Author change information

Given/first name(s)	
Family/last name	
Email address	@
Institution	

Change(s) requested for this author ☐ Remove author ☐ Add new author ☐ Make Corresponding Author

Author's Individual contributions (required for author additions only) see CRediT Contributor Roles Taxonomy

☐ Conceptualization	☐ Data curation	☐ Formal analysis
☐ Funding acquisition	☐ Investigation	☐ Methodology
☐ Project administration	☐ Resources	☐ Software
☐ Supervision	☐ Validation	☐ Visualization
☐ Writing – original draft	☐ Writing – review & editing	

Reason for this change

2.4 Author change information

Given/first name(s)	
Family/last name	
Email address	@
Institution	

Change(s) requested for this author ☐ Remove author ☐ Add new author ☐ Make Corresponding Author

Author's Individual contributions (required for author additions only) see CRediT Contributor Roles Taxonomy

☐ Conceptualization	☐ Data curation	☐ Formal analysis
☐ Funding acquisition	☐ Investigation	☐ Methodology
☐ Project administration	☐ Resources	☐ Software
☐ Supervision	☐ Validation	☐ Visualization
☐ Writing – original draft	☐ Writing – review & editing	

Reason for this change

2.5 Author change information

Given/first name(s)	
Family/last name	
Email address	@
Institution	

Change(s) requested for this author ☐ Remove author ☐ Add new author ☐ Make Corresponding Author

Author's Individual contributions (required for author additions only) see CRediT Contributor Roles Taxonomy

☐ Conceptualization	☐ Data curation	☐ Formal analysis
☐ Funding acquisition	☐ Investigation	☐ Methodology
☐ Project administration	☐ Resources	☐ Software
☐ Supervision	☐ Validation	☐ Visualization
☐ Writing – original draft	☐ Writing – review & editing	

Reason for this change

** If you have more than 5 author changes, add additional pages of the above template to provide their details.

Part 3. Confirm author order and agreement

1. Listing removed and proposed authors

Please enter the full name and details for all the removed authors in the table below, "**Agreement of removed authors**."

In the next table, "**Proposed author list**," enter the full name and details of all the proposed authors *in the order that they should appear* in the publication.

"**Full name**" is the author's name as it appears in the submission system author list.

2. Gathering signatures of all authors

This form must be signed individually by every author, including both added and removed authors. The only exception is for cases where consortia group authorship was declared at submission, in which case the corresponding author may sign on behalf of the group.

While handwritten signatures are acceptable, we highly encourage the use of electronic signature software (DocuSign, Adobe Sign, Dropbox Sign, or similar) with valid e-signatures. These digital signatures should reflect your institutional information and email as provided in the author list below.

By signing this form all authors agree:

1) that they have read and acknowledge the publishing ethics policies linked in the "Important Information" section of this form, and;

2) to the addition and/or removal of the authors listed in section 2 and to the revised order of the author list in this section 3, and;

3) that all information provided accurately reflects the authorship of the article.

Agreement of removed authors

Full name	Email address	Signature	Date
	@		
	@		
	@		
	@		
	@		

Add additional page(s) if needed.

Proposed author list

Order	Full name	Email address	Signature	Date
01	Dr. Sharda Yashwant Salunkh	shardasalunkhe@sitcoe.orç		12-08-2026
02	Kuldeep Vayadande	kuldeep.vayadande@gmail		12-08-2020
03	Hridaynath P Khandagale	khandagalehp@gmail.com		12-08-2026
04	Pavitha Nooji	pavitha.nooji@vupune.ac.in		12-08-2026
05	Mangesh Hajare	mangesh.hajare198@gmail		12-08-2026
06	Krishna Prakash	kkp.prakash@manipal.edu		12-08-2026
07	Rakhi Bhardwaj	rakhi.bharadwaj@vit.edu		12-08-2026
08	Manasi Phand	manasiphand2005@gmail.c		12-08-2026
09	Anish Katariya	anishkatariya2011@gmail.c		12-08-2026
10	Ninad Kale	ninad.kale200@gmail.com		12-08-2026
11	Amey Kharade	ameykharade04@gmail.co		12-08-2026
12		@		
13		@		
14		@		
15		@		
16		@		
17		@		
18		@		
19		@		
20		@		
21		@		
22		@		
23		@		
24		@		
25		@		

Add additional page(s) if needed.
